# Development of a multicellular in vitro model of the meningeal blood-CSF barrier to study *Neisseria meningitidis* infection

**DOI:** 10.1186/s12987-022-00379-z

**Published:** 2022-10-26

**Authors:** Leo M. Endres, Marvin Jungblut, Mustafa Divyapicigil, Markus Sauer, Christian Stigloher, Myron Christodoulides, Brandon J. Kim, Alexandra Schubert-Unkmeir

**Affiliations:** 1 Institute for Hygiene and Microbiology, University of Würzburg, Josef-Schneider-Strasse 2, 97080 Würzburg, Germany; 2 Department of Biotechnology and Biophysics, Biocenter, University of Würzburg, Würzburg, Germany; 3 Department of Biological Sciences, University of Alabama, Tuscaloosa, AL USA; 4 Department of Microbiology Heersink School of Medicine, University of Alabama at Birmingham, Birmingham, AL USA; 5 Center for Convergent Biosciences & Medicine, University of Alabama, Tuscaloosa, AL USA; 6 Alabama Life Research Institute, University of Alabama, Tuscaloosa, AL USA; 7 Imaging Core Facility, Biocenter, University of Würzburg, Würzburg, Germany; 8 Molecular Microbiology, School of Clinical and Experimental Sciences, University of Southampton Faculty of Medicine, Southampton, UK

**Keywords:** Brain endothelial cells, Leptomeningeal cells, *Neisseria meningitidis*, Induced pluripotent stem cells, Meningeal blood-CSF barrier, Bacterial meningitis

## Abstract

**Background:**

Bacterial meningitis is a life-threatening disease that occurs when pathogens such as *Neisseria meningitidis* cross the meningeal blood cerebrospinal fluid barrier (mBCSFB) and infect the meninges. Due to the human-specific nature of *N. meningitidis*, previous research investigating this complex host–pathogen interaction has mostly been done in vitro using immortalized brain endothelial cells (BECs) alone, which often do not retain relevant barrier properties in culture. Here, we developed physiologically relevant mBCSFB models using BECs in co-culture with leptomeningeal cells (LMCs) to examine *N. meningitidis* interaction.

**Methods:**

We used BEC-like cells derived from induced pluripotent stem cells (iBECs) or hCMEC/D3 cells in co-culture with LMCs derived from tumor biopsies. We employed TEM and structured illumination microscopy to characterize the models as well as bacterial interaction. We measured TEER and sodium fluorescein (NaF) permeability to determine barrier tightness and integrity. We then analyzed bacterial adherence and penetration of the cell barrier and examined changes in host gene expression of tight junctions as well as chemokines and cytokines in response to infection.

**Results:**

Both cell types remained distinct in co-culture and iBECs showed characteristic expression of BEC markers including tight junction proteins and endothelial markers. iBEC barrier function as determined by TEER and NaF permeability was improved by LMC co-culture and remained stable for seven days. BEC response to *N. meningitidis* infection was not affected by LMC co-culture. We detected considerable amounts of BEC-adherent meningococci and a relatively small number of intracellular bacteria. Interestingly, we discovered bacteria traversing the BEC-LMC barrier within the first 24 h post-infection, when barrier integrity was still high, suggesting a transcellular route for *N. meningitidis* into the CNS. Finally, we observed deterioration of barrier properties including loss of TEER and reduced expression of cell-junction components at late time points of infection.

**Conclusions:**

Here, we report, for the first time, on co-culture of human iPSC derived BECs or hCMEC/D3 with meningioma derived LMCs and find that LMC co-culture improves barrier properties of iBECs. These novel models allow for a better understanding of *N. meningitidis* interaction at the mBCSFB in a physiologically relevant setting.

**Supplementary Information:**

The online version contains supplementary material available at 10.1186/s12987-022-00379-z.

## Background

Bacterial meningitis is a devastating disease defined by meningeal inflammation in response to bacterial infection [1, 2]. Despite vaccination efforts, the human-specific, Gram-negative bacterium *Neisseria meningitidis* (Nm, meningococcus) remains one of the leading causes of bacterial meningitis worldwide [3–5]. The pathogen asymptomatically colonizes the nasopharynx of up to 35% of the healthy population, depending on age and other risk factors [6]. *N. meningitidis* can cross the epithelial nasopharyngeal barrier, disseminate systemically via the bloodstream, and cause invasive meningococcal disease (IMD), such as septicemia and/or meningitis [7]. Although treatable with modern antibiotic therapy, systemic meningococcal infection is still associated with neurological sequalae and mortality [1, 2].

A critical step in the pathogenesis of meningococcal meningitis is the traversal of the meningeal blood-cerebrospinal fluid barrier (mBCFSB) and subsequent interaction with leptomeningeal cells (LMCs) [8, 9]. Blood-central nervous system (CNS) barriers such as the blood–brain barrier (BBB) and the mBCSFB are comprised of highly specialized brain endothelial cells (BECs) that maintain CNS homeostasis by facilitating transport of nutrients and restricting passage of toxins, drugs, and pathogens [1]. One of the major phenotypes unique to BECs compared to peripheral ECs is the presence of complex adherens and tight junctions that minimize space between adjacent cellular membranes and prevent paracellular passing of material into the CNS [10, 11]. BECs of the mBCSFB make up blood vessels that run through CSF-filled spaces between the meninges, for instance the subarachnoid space [8, 9, 12]. In contrast to the BBB where BECs are surrounded by cells of the neurovascular unit, namely astrocytes and pericytes [10, 11], BECs of the mBCSFB are surrounded by and enclosed in sheets of LMCs that make up the arachnoid and the pia mater [8].

*N. meningitidis* interaction with BECs has primarily been evaluated using immortalized ECs and BECs in vitro due to the human-specific nature of the pathogen (reviewed in [13]). Previous studies have identified several virulence factors important for adherence to and invasion of BECs such as type-IV pili (Tfp), the opacity proteins OpcA and Opa as well as a series of minor adhesion or adhesion-like proteins (e.g. adhesin complex protein, Neisserial adhesin A, *Neisseria* hia homologue A protein or the autotransporter meningococcal serine protease A). Furthermore, corresponding binding host-cell receptors such as CD147 and α5β1/αvβ3 integrins were discovered [14–22]. Effects on signaling pathways and rearrangement of cytoskeletal as well as cell-membrane and junction components in BECs have been described in detail after infection of these cells with *N. meningitidis* [14–16, 18, 23–28].

Finally, two major routes for meningococcal traversal of the BEC barrier have been proposed, the first being an intra- or transcellular pathway resulting from tight interaction of bacterial adhesins/ invasins and cellular receptors as well as vesicular uptake after formation of microvillus-like structures around the bacteria [17, 18, 27, 29–32]. Inter- or paracellular crossing by *N. meningitidis* has been suggested by several studies reporting that barrier integrity is compromised under bacterial infection due to rearrangement, degradation, or downregulation of cell-junction components [15, 25, 28, 33].

Relatively little is known about the subsequent meningococcal traversal of the LMC sheet enclosing the vessel endothelium and *N. meningitidis* interaction with LMCs of the arachnoid and pia mater. Previous studies have identified bacterial factors that contribute to *N. meningitidis*-LMC interaction such as Tfp and have characterized the inflammatory response of LMCs to infection [34–37]. In these studies, LMCs derived from meningiomas were used, which have been validated for infection studies with *N. meningitidis* and other bacterial pathogens of the CNS [8, 34–39].

Up to this point, studies of *N. meningitidis* interaction at the mBCSFB have primarily utilized monocultures of immortalized BECs, which do not retain critical BBB phenotypes such as high barrier tightness [11, 40, 41]. Although some studies show that co-culture with other cell types of the CNS was able to improve barrier phenotype, defining measurables such as transendothelial electrical resistance (TEER) remained relatively low [11, 40, 41]. The immortalized microvascular endothelial cell line hCMEC/D3 is a robust and widely utilized in vitro model to study *N. meningitidis*-BEC interaction, although can lack some key BEC phenotypes [14–16, 42, 43].

Recent advances in stem-cell technologies have generated model BEC-like cells derived from human induced pluripotent stem cells (iPSCs) that better reflect the barrier phenotype of BECs [11, 44, 45]. iPSC derived BECs (iBECs) show characteristic expression of BBB markers such as adherens and tight junction components, exhibit high TEER and respond to co-culture with other cell types of the CNS [11, 40, 44–49]. Recently, iBEC monoculture models have been validated for infection studies with meningeal pathogens such as *Streptococcus agalactiae* (group B streptococcus) [50–52], and we have begun to use iBECs monoculture models to study their interaction with *N. meningitidis* [33, 46]. Additionally, this model has also been utilized to examine viral pathogens with neurotropism [53, 54]. However, to our knowledge, effects of co-culture with other CNS cell types on host–pathogen interaction at the mBCSFB has not yet been evaluated.

Here, we report on the development of a physiologically relevant in vitro model of the human mBCSFB using BECs derived from induced pluripotent stem cells in co-culture with meningioma-derived LMCs to examine *N. meningitidis* interaction. In parallel, we developed BEC-LMC co-culture models using the established infection model cell line hCMEC/D3 for reference.

## Methods

### Cell culture

Human induced pluripotent stem cell (iPSC) line IMR90-4 (WiCell) was maintained on Matrigel (Corning) coated 6-well plates (Sarstedt) in StemFlex medium (Gibco), passaging twice a week and changing medium daily as previously described [33, 44–46, 48]. Meningioma cells of the meningothelial histological subtype were derived from tumor biopsies and characterized as previously described [36]. Meningioma cells were grown in DMEM + GlutaMAX (Gibco) with 10% FCS (Gibco) and 1% Pen/Strep (Gibco) and passaged twice a week with a seeding density of 4 × 10^3^ cells/cm^2^. The cells were expanded and used for experiments up to passage 10 or until showing signs of senescence. hCMEC/D3 cells (Sigma, SCC066) were cultured in EndoGRO-MV Complete Culture Media (Sigma) splitting as needed. hCMEC/D3 and meningioma cells were grown on 30 µg/ml collagen 1 (rat tail, Gibco) coated tissue culture flasks.

### Generation of brain endothelial-like cells from iPSCs

iPSC-derived brain microvascular endothelial-like cells (iBECs) were differentiated as previously described [33, 44–46, 48]. Briefly, iPSCs were seeded from a single cell suspension onto Matrigel coated cell culture flasks (Sarstedt) at a density of 1 × 10^4^ cells/cm2 and expanded in StemFlex medium for 3 days with daily medium changes. Following iPSC expansion, the cells were differentiated in unconditioned medium [UM; DMEM/F-12 (Gibco), 20% Knockout serum replacement (Gibco), 1% minimal essential medium-nonessential amino acids (Gibco), 0.5% GlutaMAX (Gibco), and 0.07% beta-mercaptoethanol (Sigma)] for 6 days, changing medium daily. After 6 days, the medium was changed to basic EC medium [human endothelial cell serum-free media (Gibco) and 1% platelet-poor plasma derived serum (Fisher) or 1% B-27 supplement (Gibco)], supplemented with 20 ng/ml basic fibroblast growth factor (bFGF, ReproTech), and 10 μM all trans-retinoic acid (RA, Sigma), for 2 days. Finally, the differentiated iBECs were purified onto collagen IV (Sigma) and Fibronectin (Sigma) coated plates and transwells inserts (Corning; Greiner). Basic fibroblast growth factor and retinoic acid were removed one day after iBEC purification. Quality of differentiated iBECs was assessed via TEER measurements and immunofluorescence staining of characteristic BEC markers.

### Co-culture of BECs and leptomeningeal cells

On day 8 of iBEC differentiation, meningioma cells were seeded at a density of 3.6 × 10^4^ cells/cm^2^ on the underside of transwell inserts [0.4 µm inserts (Corning), or 3 µm inserts (Greiner)] coated with collagen IV and fibronectin on top, and 150 µg/ml collagen 1 (rat tail, Gibco) on the bottom (direct co-culture). For indirect co-culture, 2.2 × 10^4^ cells/cm^2^ were seeded onto collagen 1-coated cell culture plates, to which collagen IV and fibronectin-coated transwell inserts were added later. After 4 h of incubation at 37 °C and 5% CO_2_, transwell inserts were flipped into the designated wells and iBECs were purified on top of the transwell membrane, seeding differentiated cells at a density of approximately 9 × 10^5^ cells/cm^2^ onto the collagen IV and fibronectin matrix. Co-cultures were cultivated in EC medium with Pen/Strep for 1 day, before changing media to basic EC medium without bFGF and RA. Experiments were conducted on day 2 of co-culture (i.e., day 10 of the iBEC differentiation protocol). For co-culture of hCMEC/D3 cells and meningioma cells, transwell inserts were coated entirely with 150 µg/ml collagen 1. Meningioma cells were seeded as described for the iBEC model. After 4 h, hCMEC/D3 cells were seeded on top of the membrane at a density of 9 × 10^4^ cells/cm2. Co-cultures were cultivated for 3 days in EndoGRO medium before performing experiments.

### Bacterial strains

*Neisseria meningitidis* serogroup B strain MC58—sequence type (ST)-74 [ST-32 clonal complex (cc)], kindly provided by E. R. Moxon [55]—was used in this study. Meningococci were grown on Columbia agar with 5% sheep blood (bioMérieux) at 37 °C and 5% CO_2_ overnight. The following day, bacteria were subcultured in liquid proteose peptone medium (PPM)—reshly supplemented with 1% Kellogg’s supplement, 10 nM MgCL_2_ and 10 nM NaHCO_3_ (ROTH)—at 37 °C and 200 rpm for 60 to 90 min.

### Infection assays

One day before conducting infection experiments, antibiotics were removed from the cellular co-culture medium by washing twice with Dulbecco's phosphate-buffered saline (DPBS, Gibco). Medium was changed once on the day of infection, at least 1 h before adding bacteria. Bacterial infection of cells was prepared as described previously [33, 46, 56]. Bacteria grown in liquid subculture were spun down, washed in DPBS, and diluted in cell culture medium prior to infection. A multiplicity of infection (MOI) of 10 was used unless specifically noted otherwise.

### Gentamicin protection assay

Bacterial adherence and invasion of the model endothelial cell layer was evaluated using gentamicin protection assays. BEC layers were infected with *N. meningitidis* strain MC58 for 2 h or 8 h using an MOI of 100, which is within the range of MOIs used in previously published studies to investigate the interaction of the pathogen with BECs [15, 16, 24, 32, 57]. For enumeration of intracellular CFU per monolayer at the indicated times post-infection, cells were washed once with DPBS and incubated in cell culture medium with 200 µg/ml Gentamicin (Biochrom) for an additional 2 h. To evaluate the total number of cell-associated bacteria, samples were processed immediately at the indicated time points. Briefly, the cells were dissociated and lysed with 0.05% Trypsin–EDTA (Gibco) at 37 °C for 5 min and 1% saponin (SERVA) for 15 min at room temperature (RT). Samples were plated in a dilution series and numbers of bacteria were calculated from CFU counts of countable dilutions. Data are presented as total CFU recovered/monolayer.

### Transmigration assay

Rate of bacterial transmigration was determined by enumeration of CFU that had migrated from the apical to the basolateral compartment of the models within an hour after each indicated time point, using similar methodology as described previously [33]. Transwell inserts with a 3 µm pore size (Greiner) were used to ensure meningococci could pass through the membrane. Inserts were washed twice in PBS and transferred to fresh medium, only replacing the medium on the bottom. After 1 h of incubation at 37 °C and 5% CO_2_, samples from the basolateral medium were plated in a dilution series to determine bacterial loads.

### TEER and permeability experiments

Barrier tightness was assessed via transendothelial electrical resistance (TEER) using a volt-ohm meter (Millicell ERS-2, Merck) and sodium fluorescein (NaF) permeability measurements as described previously [48]. Briefly, medium on top of the transwell inserts was replaced with 10 µM NaF (Sigma) and samples were taken from the basolateral medium every 15 min for 1 h. At the final time point, an additional sample was collected from the top compartment, all samples were analyzed in a fluorescence plate reader (Tecan), and NaF permeability (Pe) was calculated as described previously [48].

### Immunofluorescence

Immunofluorescence staining of co-culture models was adapted from previously described protocols for iBEC and LMC staining [36, 46, 48]. Cells on transwell inserts were fixed in ice-cold methanol for 15 min, washed with DPBS, and incubated in 10% FCS for 1 h at RT. For detection of epithelial membrane antigen (EMA/Mucin 1) and E-Cadherin, cells were fixed in 4% paraformaldehyde and blocked with 10% FCS containing 0.1% saponin. Transwell membranes were cut out using a scalpel and transferred to wells of a cell culture plate for primary antibody staining at 4 °C overnight (Table 1). The following day, samples were washed in DPBS and secondary staining was conducted for 1 h at RT using Alexa Fluor 488 goat anti-mouse (Invitrogen, ref. A11001), and Alexa Fluor 555 donkey anti-rabbit (Invitrogen, ref. A31572) antibodies at a dilution of 1:200 in 10% FCS. After final washing steps and DAPI staining (Invitrogen, ref. D1306; 1:5000 in DPBS), membranes were mounted on glass slides (mounting medium: Fluoroshield, Sigma) and cellular staining was visualized on an Eclipse T*i*2 confocal microscope (Nikon). Image acquisition and analysis was performed using NIS Elements image software version 5.02 (Nikon). Junction coverage was determined for occludin expression using the JAnaP junction analyzer program [58].

**Table 1:** Primary antibodies used for immunofluorescence

Target antigen	Dilution	Species and clonal information	Vendor
CD31	1:200	Rb, polyclonal, ref. ab32457	Abcam
VE-Cadherin^a^	1:25	Ms, clone BV9	Santa Cruz
ZO-1^b^	1:100	Ms, clone ZO1-1A12	Invitrogen
Occludin^a^	1:200	Ms, clone OC-3F10	Invitrogen
Claudin-5^a^	1:50	Ms, clone 4C3C2	Invitrogen
Vimentin	1:100	Ms, clone V9	Invitrogen
Desmoplakin I/II	1:50	Ms, clone A-1	Santa Cruz
EMA (Mucin 1)	1:25	Ms, clone VU4H5	Santa Cruz
E-Cadherin	1:100	Ms, clone G-10	Santa Cruz
Laminin	1:50	Rb, polyclonal, ref. PA1-16730	Invitrogen

^a^Stebbins et at. 2016 [48]

^b^Kim et al. 2017 [50]

### Super resolution microscopy

After 24 h of infection with GFP-expressing wildtype strain MC58 [26], iBEC-LMC as well as hCMEC/D3-LMC in vitro co-culture models were washed once in DPBS, permeabilized in cytoskeleton buffer (10 mM MES, 150 mM NaCl, 5 mM EGTA, 5 mM glucose, and 5 mM MgCl_2_ adjusted to pH 6.11) with 0.25% glutaraldehyde (Sigma) and 0.25% Triton X-100 (ROTH) for 1–2 min at 37 °C, and fixed in cytoskeleton buffer with 2% glutaraldehyde for 10 min at RT. Samples were washed twice and quenched in 0.1% sodium borohydride (Sigma) in PBS for 7 min at RT. Following a third washing step, transwell membranes were cut out and stained with 5 U/ml phalloidin-Alexa Fluor 543 (Invitrogen, ref. A22283) or 100 nM phalloidin-ATTO 643 (ATTO-Tec, ref. AD643-81) overnight at 4 °C. The next day, samples were stained with DAPI (Invitrogen, ref. D1306; 1:5000 in DPBS) and washed in ddH_2_O. Transwell membranes were cut into two halves and mounted in ProLong^™^ Glass Antifade Mountant (TermoFisher) on clean high precision coverglass slides (BRAND) one half of the membrane with the endothelial cell side facing the coverslip the other with the meningeal cell side facing the coverslip. Mounted samples were cured at RT overnight, then stored at 4 °C.

Structured illumination microscopy (SIM) was performed on a Zeiss Elyra 7 with Lattice SIM^2^ using either a 40x (Plan-Apochromat 40x/1.4 Oil DIC M27) or a 63x (Plan-Apochromat 63x/1.4 Oil DIC M27) oil immersion objective and four different excitation lasers (405 nm diode, 488 nm OPSL, 561 nm OPSL, and 642 nm diode laser). Z-stacks of endothelial or meningeal cell layers were captured in 3D Leap mode with optimal z-steps, controlled by a piezo stage, using appropriate band-pass and long-pass filters sets. SIM imaging was performed with 9 or 13 phase-shifts of the lattice SIM pattern. Reconstruction of the super-resolved images was performed using ZEN 3.0 SR FP2 (black) (Version 16.0.10.306; Zeiss) with SIM and SIM^2^ processing modules. Final images were processed using Imaris 9.2.1. (Bitplane).

### Transmission electron microscopy

Samples were prepared for transmission electron microscopy (TEM) at the indicated time points post-infection. Transwell membranes were washed in DPBS and fixed in 2.5% glutaraldehyde (Sigma) for 1 h at RT, washed three times in 50 mM cacodylate buffer (Roth), cut out using a scalpel and transferred to a glass vial containing cacodylate buffer for further processing. Samples were processed for EM as previously published [59], with the following modification: samples were infiltrated and embedded in Durcupan (Sigma). Electron micrographs were recorded on a JEM-1400Flash transmission electron microscope (JEOL) equipped with a Matataki camera system.

### RNA extraction and quantitative PCR

After the indicated time of incubation with bacteria, cells from transwell models were collected using Accutase (Sigma) dissociation for 15 min (iBEC) or 5–7 min (hCMEC/D3). Uninfected cells were used as control. Cell lysis and RNA purification was performed using a NucleoSpin RNA kit (Machery-Nagel). cDNA was generated using LunaScript RT (New England BioLabs). Quantitative PCR (qPCR) was conducted on a StepOnePlus real-time PCR thermocycler (Applied Biosystems) using PowerUp SYBR Green master mix (Applied Biosystems). Primer sequences are supplied in Table 2. Primers were validated by primer efficiency analysis and agarose gel electrophoresis of the qPCR product. qPCR data are presented as fold change over *18S* using the cycle threshold (ΔΔC_t_) calculation [60].

**Table 2:** Primers used for qPCR

Gene	Forward sequence	Reverse sequence
*18S rRNA*^*a*^	GTAACCCGTTGAACCCCATT	CCATCCAATCGGTAGTAGCG
*PECAM1*	AAGTGGAGTCCAGCCGCATATC	ATGGAGCAGGACAGGTTCAGTC
*CDH5*	AAGGACATAACACCACGAAACG	CAAACTGCCCATACTTGACTGTG
*TJP1*	GTCCAGAATCTCGGAAAAGTGCC	CTTTCAGCGCACCATACCAACC
*OCLN*	ATGGCAAAGTGAATGACAAGCGG	CTGTAACGAGGCTGCCTGAAGT
*CLDN5*	CTCTGCTGGTTCGCCAACAT	CAGCTCGTACTTCTGCGACA
*SNAI1*^*b*^	GGACCCACACTGGCGAGAAG	ATTCGGGAGAAGGTCCGAGC
*CXCL8*^*c*^	AGCTCTGTGTGAAGGTGCAG	AATTTCTGTGTTGGCGCAGT
*CXCL1*^*d*^	CTCTTCCGCTCCTCTCACAG	GGGGACTTCACGTTCACACT
*CXCL2*^*d*^	CTCAAGAATGGGCAGAAAGC	AAACACATTAGGCGCAATCC
*IL6*^*d*^	GGAGACTTGCCTGGTGAAAA	CAGGGGTGGTTATTGCATCT
*CCL20*^*d*^	GCGCAAATCCAAAACAGACT	CAAGTCCAGTGAGGCACAAA

^a^Rho et al. 2010 [61]

^b^Han et al. 2011 [62]

^c^Kim et al. 2019 [51]

^d^van Sorge et al. 2008 [63]

### SNAI1 knockdown experiment

hCMEC/D3 cells were transfected with siRNA using TransIT-siQUEST^®^ Transfection Reagent (Mirus) according to manufacturers instructions. hCMEC/D3 were seeded at a density of 2 × 10^5^ cells/well onto a 24-well plate on the day prior to transfection. *SNAI1* siRNA (Santa Cruz) or scrambled, FITC conjugated siRNA (Santa Cruz) was added at a final concentration of 50 nM to a mix of EndoGRO basal medium (50 µl/well) and TransIT-siQUEST® transfection reagent (1.5 µl/well) and incubated for 30 min at RT before transfection. After 24 h of transfection, the medium was changed, and the cell layers were infected with *Neisseria meningitidis* serogroup B strain MC58 at an MOI of 10 for 24 h. Finally, cell lysates were collected for qPCR analysis. To assess cell viability, whole cells were collected, washed in PBS, and stained with 1 mg/ml Propidium iodide (PI) for 10 min before flow cytometry analysis. Non-transfected cells treated with or without 2.5% Triton-X during PI staining were used as controls.

### IL-8 ELISA

After the indicated time of incubation with bacteria, supernatants were collected from the transwell models and used for detection of IL-8 using the Human IL-8 ELISA Set (BD Biosciences) and following the manufacturers instructions. Briefly, 96-well plates (clear, flat bottom) were coated with capture antibody (1:250 in 0.1 M sodium carbonate, pH 9.5) at 4 °C o/n. After blocking with 10% FCS for 1 h at RT, samples and standard were diluted appropriately in 10% FCS and added to the plate in technical duplicate. After 2 h of incubation at RT in the sealed plate, a 1:1 mix of detection antibody and Streptavidin–horseradish peroxidase conjugate (both 1:250 in 10% FCS) was added for 1 h at RT. Between all mentioned steps, multiple washes were performed using 0.05% Tween in PBS. Finally, the wells were incubated with tetramethylbenzidine and hydrogen peroxide (Thermo Fisher) at a 1:1 ratio for 15–30 min at RT, before the reaction was stopped with 2 N H_2_SO_4_. Wavelength corrected absorbance (450 nm–570 nm) was measured using an absorbance plate reader (Tecan) and protein concentrations were calculated using a standard curve.

### Statistics

GraphPad Prism version 6.01 (GraphPad Software Inc.) was used for all statistical analysis. 2-tailed Student’s t test was used for pairwise comparison, where appropriate. Analysis of variance (ANOVA) followed by Dunnett’s multiple comparisons test was used for multiple comparisons. Statistical significance was accepted at a P value of less than 0.05.

## Results

### Development and characterization of mBCSFB co-culture models

**Fig. 1:**
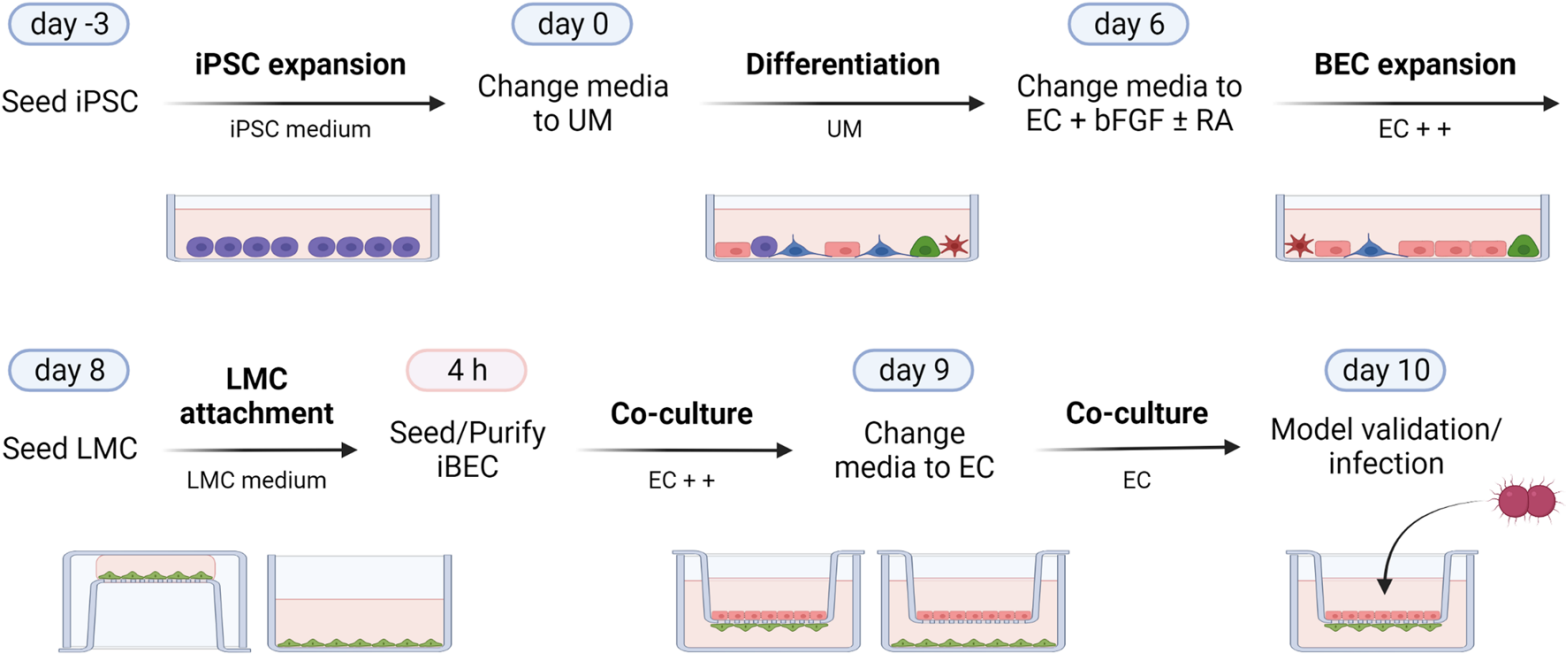
Schematic representation of iPSC-derived brain endothelial-like cell (iBEC) differentiation and co-culture with leptomeningeal cells (LMCs). iBEC differentiation as previously described [44–46, 48]. On day 8, LMC are seeded on the underside of transwells or on standard tissue culture plates. After 4 h of incubation, iBECs are purified onto the apical side of the transwells. Co-cultures were cultivated in EC medium with Pen/Strep for 1 day, before removal of factors added to the basal EC medium. Experiments were conducted on day 10 of differentiation (day 2 of co-culture). Figure created with BioRender.com

**Fig. 2:**
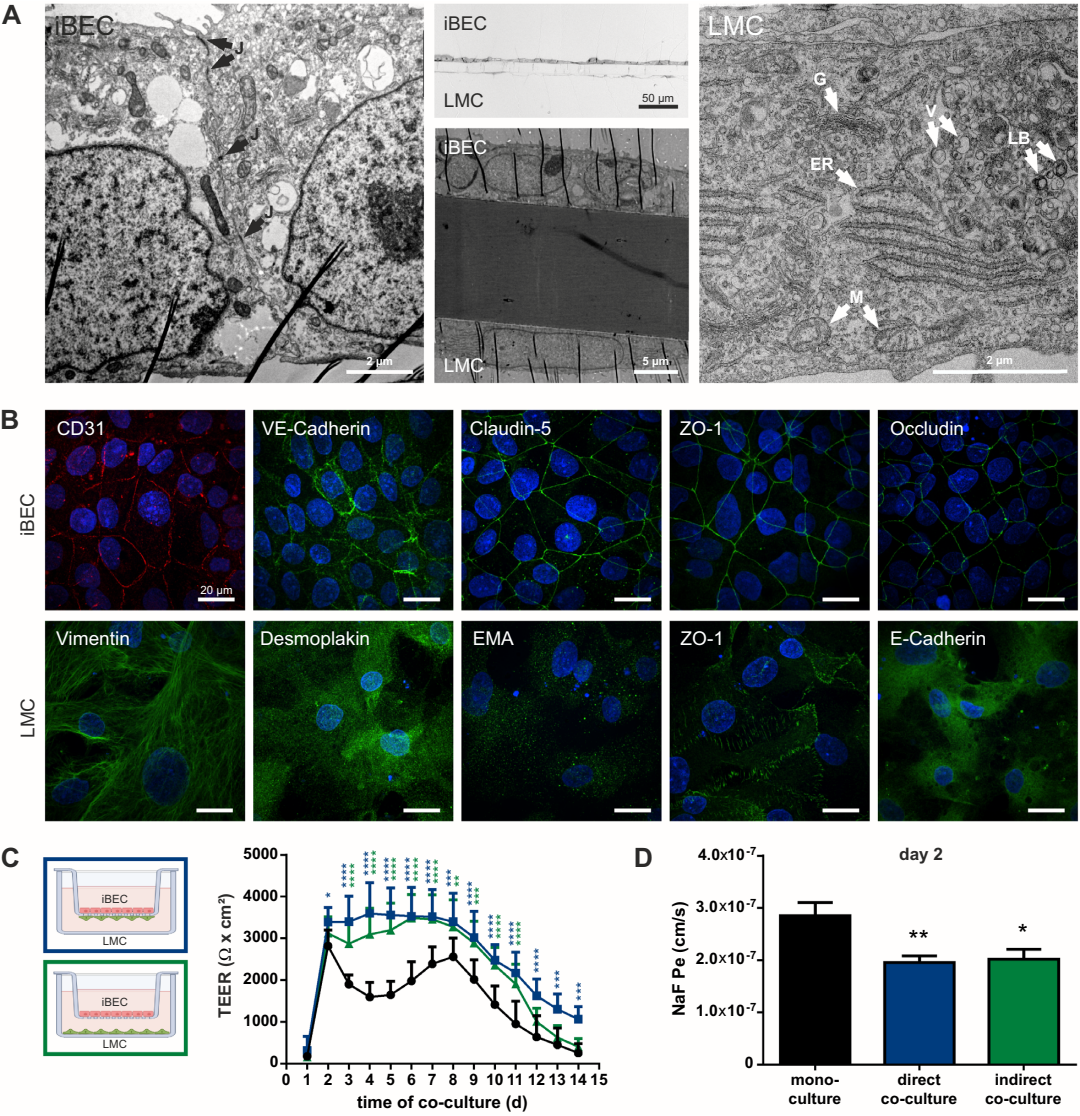
Characterization of the iBEC-LMC co-culture model. **a** Transmission electron microscopy (TEM) of iBECs and LMCs co-cultured on transwell for 2 days. A widefield image of a semithin cross-section of the embedded model (middle, top) is presented in addition to electron micrographs of ultrathin sections. Labeled structures: cell junctions (J), Golgi apparatus (G), rough endoplasmic reticulum (ER), mitochondria (M), vesicles (V), lamellar bodies (LB). **b** Immunofluorescence staining of iBEC (top) and LMC (bottom) monolayers on either side of transwell membranes for endothelial adherens junction proteins (CD31, VE-Cadherin), tight junction components (claudin-5, ZO-1, occludin, E-cadherin), and meningioma markers (vimentin, desmoplakin I/II, epithelial membrane antigen), performed after 2 days of co-culture. Nucleus staining with DAPI (blue). Scale bars represent 20 µm. **c** Transendothelial electrical resistance (TEER) of iBEC monoculture (black), direct (blue) and indirect (green) iBEC-LMC co-culture over a time-course of 14 days. **d** Sodium fluorescein permeability (NaF Pe) of iBEC mono and co-culture models on day 2 of co-culture, determined according to previously describe protocols [48]. All data presented as mean ± SD from three independent experiments and iBEC differentiations performed in triplicate (n = 9). *p < 0.05, **p < 0.01, ***p < 0.001, **** p < 0.0001; ANOVA followed by Dunnett’s multiple comparisons test; direct (blue) and indirect (green) co-culture vs monoculture

Brain endothelial-like cells (iBECs) were differentiated from iPSC line IMR90-4 according to previously describe protocols [44–46, 48] and co-cultured with model leptomeningeal cells, derived from meningioma [36], (LMCs) for two days immediately upon purification (Fig. 1). The co-culture models were characterized using transmission electron microscopy (TEM) and confocal immunofluorescence imaging. At the optimized seeding densities, TEM and imaging of semithin cross-sections revealed morphologically distinct monolayers of iBECs and LMCs on either side of the transwell membrane (Fig. 2a). Using TEM, we observed various electron dense regions between adjacent cells of the dense iBEC monolayer, most likely representing cellular junctions including tight junctions, adherens junctions, and desmosomes (Fig. 2a). The much larger LMCs on the basolateral side displayed a spread morphology with long cellular processes extending out to other cells, and cytosolic components including Golgi, endoplasmic reticulum, vesicles, lamellar bodies, and mitochondria could be visualized via TEM (Fig. 2a). Immunofluorescence staining showed characteristic expression of brain endothelial markers such as CD31, VE-cadherin, and tight junction proteins ZO-1, occludin, and claudin-5 at the cell junctions within the iBEC layer (Fig. 2b). These markers were not observed in the LMC layer apart from a distinct expression of ZO-1 (Fig. 2b; Additional file 1: Video S1). The meningioma derived cells of the meningothelial subtype retained their characteristic morphology and expression of histopathological markers vimentin, desmoplakin, and epithelial membrane antigen (EMA) in co-culture (Fig. 2b). Despite immunopositivity for E-Cadherin, which is reported to be expressed in arachnoid barrier forming leptomeningeal cells [64], these cells did not show junctional localization of the protein. Finally, as current research has provided insight into the importance of the extracellular matrix on BBB development and maturation, particularly focusing on laminin [65], we have staining the iBEC-LMC co-culture model for this class of ECM proteins and found expression on both sides of the transwell membrane (Additional file 2: Fig. S1). Together these results demonstrate that iBECs can be co-cultured with the LMC cells, and staining patterns suggests that both cell types retain their characteristics and remain distinct in culture.

For reference, BEC-LMC co-culture models using the established BEC cell line hCMEC/D3 [42, 43] were developed in parallel and characterized using the same methods. After 3 days of co-culture, confluent layers of both cell types were observed on either side of the transwell membrane and expression of BEC makers such as VE-Cadherin and ZO-1 was detected (Additional file 2: Fig. S2a, b). However, within the hCMEC/D3 layer, we were unable to achieve clear and uniform immunofluorescence staining of junctional BEC markers at the cell–cell borders (Additional file 2: Fig. S2b). While hCMEC/D3s can reportedly lack certain barrier properties such as continuous junctional expression of tight junction components, the model does retain much of the BEC phenotype, especially compared to other human primary or immortalized cell lines, and has been widely used to study *N. meningitidis*-BEC interaction in vitro [11, 14–16, 28, 41–43]. Therefore, we continued to use hCMEC/D3s for reference in this study, reporting on hCMEC/D3 co-culture with LMCs for the first time.

### LMC co-culture increases BEC barrier tightness and stability

To evaluate effects of LMC co-culture on barrier tightness and integrity of the iBEC model, we measured transendothelial electrical resistance (TEER) and sodium fluorescein (NaF) permeability. TEER measurements were conducted over a period of 14 days after iBEC purification and start of co-culture (without media changes after the switch to EC medium on day 1; i.e., day 9 of differentiation) (Fig. 2c) and NaF permeability was determined on indicated days (Fig. 2d and Additional file 2: Fig. S3b). Both, iBEC monoculture and iBEC-LMC co-culture reached TEER levels of > 2000 Ω x cm^2^ on day 2 of co-culture [40] (Fig. 2c), which corresponds to day 10 of the iBEC differentiation protocol when iBEC monoculture typically reaches peak TEER [46, 48]. At that time, TEER was increased (3389 ± 354 Ω x cm^2^) and NaF permeability was reduced (1.96 ± 0.38 × 10^–7^ cm/s) in the iBEC-LMC direct co-culture models, compared to iBEC monoculture (2820 ± 384 Ω x cm^2^; 2.85 ± 0.73 × 10^–7^ cm/s) (Fig. 2c, d). After that, TEER remained stable and significantly higher in both direct and indirect co-culture for 7 days (Fig. 2c). LMC cells alone, cultured on the transwell underside, did not generate TEER higher than 37 ± 17 Ω x cm^2^, and the increase in TEER continued to be accompanied by a reduction in permeability to NaF (day 4; Additional file 2: Fig. S3). TEER of iBEC monoculture dropped after peaking on day 2 before rising again to near peak levels and, finally, continuously descending after day 8 (Fig. 2c), which is comparable to previously reported data [66]. Continuous reduction of TEER beginning on day 8 was also observed in both co-culture models in parallel to monoculture. However, while TEER reached near-monoculture levels in indirect co-culture on day 12, TEER remained significantly higher with direct co-culture for the remainder of the experiment (day 14; Fig. 2c). In our hCMEC/D3 based models, peak TEER was reached after 3–4 days of co-culture and did not exceed 30 ± 8 Ω x cm^2^ within 14 days (Additional file 2: Fig. S2c), remaining substantially below physiological range [40]. Permeability to NaF was measured at 2.25 ± 0.36 × 10^–5^ cm/s for direct co-culture (Additional file 2: Fig. S2d). Elevated TEER was also detected in hCMEC/D3-LMC direct co-culture compared to hCMEC/D3 monoculture, accompanied by lower NaF permeability (Additional file 2: Fig. S2d). However, the maximum TEER was comparable to TEER from LMC cells alone (37 ± 17 Ω x cm^2^; Additional file 2: Fig. S3a). In summary, LMC co-culture increased BEC barrier tightness and stability over time as reflected by lower NaF permeability and higher TEER. This effect was most pronounced in the high TEER iBEC models and was observed in both direct and indirect co-cultures, suggesting that LMC co-culture improves the barrier function of iBEC monolayers.

### Neisseria meningitidis interaction with BECs with LMC co-culture

**Fig. 3:**
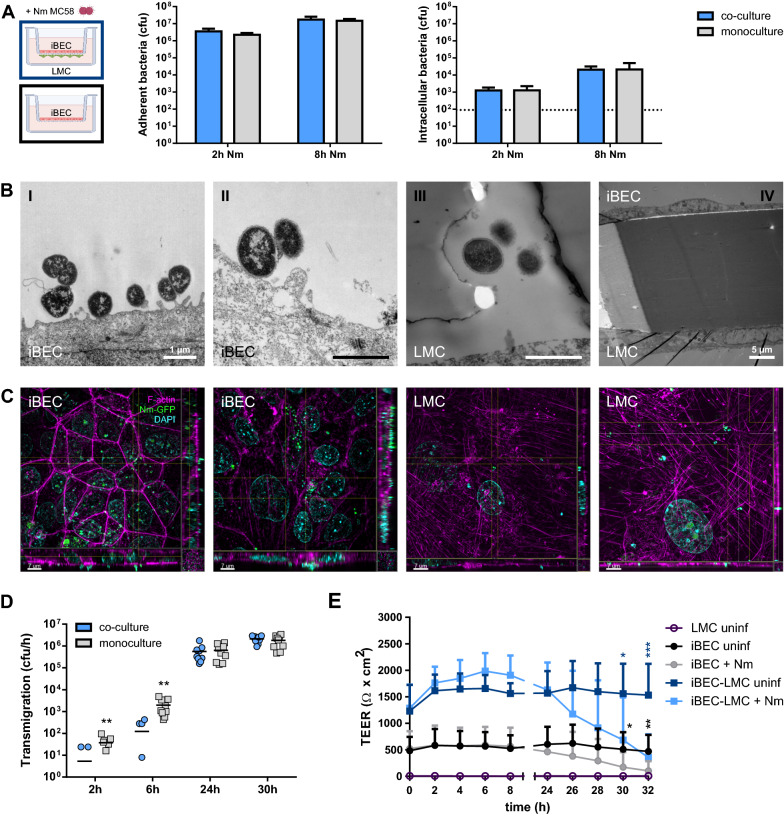
*N. meningitidis* interaction with the iBEC-LMC direct co-culture model. Models were infected from the iBEC side (MOI = 10, unless specified otherwise) on day 2 of co-culture for the times indicated. **a** Enumeration of adherent and intracellular cfu/monolayer on iBEC layers with or without LMC co-culture after infection with *N. meningitidis* at an MOI of 100, determined by gentamicin protection assays. **b** TEM of iBEC-LMC co-culture models infected with *N. meningitidis* for 6 h (**I**), 24 h (**II**), and 30 h (**III, IV**). Scale bars represent 1 µm, unless labeled otherwise. **c** Structured illumination microscopy showing iBEC or LMC layers from iBEC-LMC co-culture, stained for f-actin (phalloidin-546, magenta) and DNA (DAPI, blue) after 24 h of infection with a GFP expressing *N. meningitidis* strain. Images presented as maximum intensity projection in Z and including maximum intensity projections in X and Y (orthoslices) as indicated by the crosshairs. Scale bars represent 7 µm. **d** *N. meningitidis* transmigration rates determined by enumeration of cfu in the basolateral compartment after 1 h of incubation in fresh basolateral media following the indicated infection time points. **e** TEER of infected and uninfected iBEC monoculture and iBEC-LMC co-culture over a time-course of 32 h post-infection. Data presented as mean ± SD from three independent experiments and iBEC differentiations performed in duplicate (**a**, n = 6) or triplicate (**d**, **e**; n = 9). *p < 0.05, **p < 0.01, ***p < 0.001; Student’s t test; direct co-culture (blue) vs monoculture (grey) (**d**), infected vs uninfected control (**e**)

To validate the mBCSFB co-culture models for infection studies with *N. meningitidis*, we first examined *N. meningitidis* interaction at the BEC layer. BEC layers were infected with *N. meningitidis* serogroup B strain MC58 after 2 (iBEC models) or 3 days (hCMEC/D3 models) of direct co-culture with LMCs. For comparison, infection experiments were also performed on iBEC and hCMEC/D3 monocultures. Bacterial adherence and invasion were determined 2 and 8 h post-infection (p.i.) using gentamicin protection assays. *N. meningitidis* strain MC58 significantly adhered to iBECs in iBEC-LMC co-cultures already after 2 h of infection (3.58 ± 1.53 × 10^6^ cfu/monolayer) and adherence increased marginally when infection was carried out for 8 h (1.75 ± 0.80 × 10^7^ cfu/monolayer; Fig. 3a). The number of recovered intracellular bacteria was comparatively low (1.26 ± 0.60 × 10^3^ cfu/monolayer, 2 h p.i.) and increased over time (2.06 ± 1.12 × 10^4^ cfu/monolayer, 8 h p.i.). The average counts of adherent and invasive bacteria per monolayer were comparable to iBEC monoculture (Fig. 3a). Compared to iBECs, similar numbers of adherent bacteria were detected on hCMEC/D3 layers of hCMEC/D3-LMC co-culture 2 h (5.19 ± 2.25 × 10^6^ cfu/monolayer) and 8 h p.i. (1.39 ± 0.54 × 10^7^ cfu/monolayer) (Additional file 2: Fig. S4a). Counts of recovered intracellular bacteria were lower after 2 h (42 ± 33 cfu/monolayer) and comparable after 8 h of infection (4.43 ± 5.38 × 10^4^ cfu/monolayer). No significant difference in adherence or invasion was observed between hCMEC/D3-LMC co-culture and hCMEC/D3 monoculture (Additional file 2: Fig. S4a). Next, we examined the interaction between *N. meningitidis* and iBECs of the iBEC-LMC direct co-culture model using TEM. In parallel, iBECs grown in co-culture with LMC and infected with a green fluorescence protein (GFP) expressing mutant of strain MC58 for 24 h, were stained for F-actin and analyzed using structured illumination microscopy (SIM). We detected bacteria tightly associated with iBECs as well as previously described cellular structures such as cytoplasmic protrusions around the adherent bacteria [29, 57] (Fig. 3bI, II), and observed clusters of meningococci, commonly described as microcolonies (Fig. 3c, left panels). Taken together, these results demonstrate that BEC-LMC co-cultures serve as novel, suitable cellular mBCSFB models for infection studies with *N. meningitidis*.

### Neisseria meningitidis traversing the mBCSFB models

Next, we investigated the traversal of *N. meningitidis* across the mBCSFB. After infecting the BEC layers with *N. meningitidis* strain MC58, we analyzed iBEC-LMC co-culture models using TEM and SIM. Using TEM, we were able to observe few bacteria traversing the iBEC-LMC co-culture model and interacting with the LMC layer at 24 and 30 h p.i. (Fig. 3bIII, IV). SIM analysis of iBEC-LMC models, stained for F-actin after 24 h of infection with GFP expressing *N. meningitidis* strain MC58, revealed small numbers of meningococci on the apical side of or in the process of crossing the LMC layer on the basolateral side of the model (Fig. 3c, right panels). To quantify bacterial transmigration, we conducted bacterial transmigration assays on BEC-LMC co-culture and BEC monoculture models. 2, 6, 24 and 30 h p.i., bacterial transmigration rates were determined by enumeration of cfu in the basolateral compartment after 1 h of incubation in fresh basolateral media. While bacterial traversal of the iBEC-LMC model was mostly undetectable 2 h p.i., bacteria crossing the barrier were detected more frequently and in greater numbers (> 100 cfu/h) after 6 h (Fig. 3d). At 24 h p.i., transmigration rates had increased substantially (0.16–1.76 × 10^6^ cfu/h) and continued to increase over the 30 h time course of the experiment (0.94–2.92 × 10^6^ cfu/h). Similar results were obtained from infected iBEC monocultures, although measured bacterial traversal was higher at the early infection timepoints (0.43–4.84 × 10^3^ cfu/h at 6 h p.i.) (Fig. 3d). In the hCMEC/D3-LMC models, high rates of bacterial transmigration were already detected 2 h p.i. (0.02–1.08 × 10^6^ cfu/h) and increased to a lesser degree up to 6 h p.i. (1.52–5.28 × 10^6^ cfu/h) before remaining at approximately the same level (1.64–8.40 × 10^6^ cfu/h, 24 h p.i.) (Additional file 2: Fig. S4b). The numbers of bacteria traversing hCMEC/D3s grown in monoculture were comparable to hCMEC/D3-LMC co-culture data, although slightly higher at 2 h (0.06–1.8 × 10^6^ cfu/h) and slightly lower at 24 h p.i. (0.24–4.8 × 10^6^ cfu/h) (Additional file 2: Fig. S4b).

In previous research, it has been a matter of debate, whether *N. meningitidis* crosses the brain endothelium at the mBCSFB via a transcellular [17, 29, 31] or a paracellular route [25, 28] or both [33]. To gain more information on the mechanism of barrier traversal in our models, we measured TEER over a 32 h time course p.i. as an indication of barrier integrity and restriction to paracellular transport. In our iBEC-LMC model, TEER remained high for 24 h p.i. (Fig. 3e), although we detected increasing amounts of bacterial transmigration in that time frame (Fig. 3d), suggesting transcellular traversal by *N. meningitidis*. Additionally, we still observed intact cell-junctions after 24 h of infection using TEM (Fig. 3bII). However, after 24 h of infection, TEER began to drop leading to significant loss of TEER compared to the uninfected control by 30 h p.i. (Fig. 3e), which has also been previously described for iBEC monoculture [33]. We observed similar effects analyzing infected iBEC monocultures, in this study, although TEER was lower overall compared to iBEC-LMC co-culture (Fig. 3e). TEER of hCMEC/D3-LMC co-culture and hCMEC/D3 monoculture models also remained stable for up to 24 h of infection before dropping to significantly lower levels, although total TEER was substantially lower compared to the iBEC model (Additional file 2: Fig. S4c). These data indicate that, while the barrier remains intact for some time, prolonged *N. meningitidis* infection eventually compromises barrier integrity of BECs at the mBCSFB. Taken together, these results suggest a transcellular route for *N. meningitidis* traversal of the mBCSFB, although loss of barrier integrity during the later course of infection (> 24 h of infection) may also enable paracellular crossing.

### Barrier deterioration upon prolonged N. meningitidis infection

**Fig. 4:**
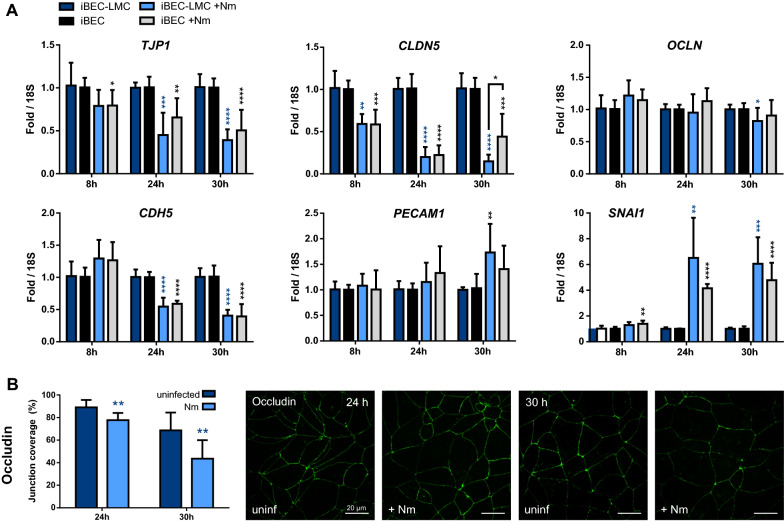
Effects of *N. meningitidis* infection on cell-junction expression in iBECs of mono- and co-culture models. **a** Relative expression of genes for endothelial adherens junction proteins CD31 (*PECAM1*) and VE-Cadherin (*CDH5*), and tight junction components ZO-1 (*TJP1*), occludin (*OCLN*), and claudin-5 (*CLDN5*) in iBECs from direct co-culture with LMCs (blue bars) and iBEC monoculture (black/gray bars), with (light bars) or without (dark bars) *N. meningitidis* infection, quantified by qPCR and normalized to 18S rRNA. Data presented as mean ± SD from four independent experiments and iBEC differentiations performed in duplicate (n = 6–8). **b** Junction coverage of occludin in iBEC monolayers from iBEC-LMC co-cultures analyzed using confocal microscopy and JAnaP junction analyzer software [58]. Data presented as mean junction coverage ± SD from three independent experiments, calculated using average junction coverage data from three images per experiment (n = 9). Representative images were chosen based on these calculations. *p < 0.05, **p < 0.01, ***p < 0.001, ****p < 0.0001; Student's t test; infected vs. uninfected control (blue/black asterisks directly over bars), mono vs co-culture (asterisks above brackets)

To further investigate the mechanisms behind barrier deterioration upon prolonged *N. meningitidis* infection, we analyzed expression of cell-junction components in our BEC-LMC co-culture models. First, we examined expression of adherens and tight junction genes in infected BECs from our BEC-LMC co-culture and BEC monoculture models. In the iBEC-LMC co-culture model, expression of genes encoding for endothelial adherens junction protein VE-Cadherin (*CDH5*) and tight junction proteins ZO-1 (*TJP1*) and claudin-5 (*CLDN5*) was downregulated in infected iBECs compared to the uninfected control, especially after 24 h and 30 h p.i. (Fig. 4a). The strongest effect was observed with *CLDN5*. Expression of CD31 (*PECAM1*) and occludin (*OCLN*) was not altered (Fig. 4a). Expression of *SNAI1*, which encodes for Snail1, a repressor of tight junction gene expression [67–71], was significantly increased with infection at the indicated time points (Fig. 4a). The same expression profiles were observed with iBEC monocultures (Fig. 4a), consistent with previously reported data on iBECs cultured in cell culture plates [33]. qPCR data from hCMEC/D3 samples grown in co-cultures with LMC are consistent and show reduced *PECAM1* expression in addition to downregulation of *CDH5*, *TJP1*, and *CLDN5* (Additional file 2: Fig. S5). Interestingly, expression of *OCLN* was increased in hCMEC/D3s with infection, and we observed a tendency towards upregulation of *TJP1* and *CLDN5* together with downregulation of *SNAI1* after 8 h of *N. meningitidis* infection. This observation could not be correlated with functional changes such as increased TEER, a phenomenon that was, however, occasionally observed at the early infection time points both in iBEC and hCMEC/D3 models (Fig. 3e; Additional file 2: Fig. S4c). Together, these data demonstrate downregulation of tight and adherens junction genes upon *N. meningitidis* infection.

To assess whether Snail1 plays a role in this mechanism, we performed siRNA knockdown of *SNAI1* and analyzed its effect on expression of cell-junction genes in hCMEC/D3 cells with and without *N. meningitidis* infection (Additional file 2: Fig. S6). *SNAI1* knockdown lead to increased expression of *CLDN5*, *CDH5*, and *PECAM1*, which are all Snail1 targets [72]. However, we observed no significant rescue effect of *N. meningitidis* induced downregulation of these genes. Together, these findings indicate that other mechanisms instead or in addition to Snail1 mediated repression may be responsible for the downregulation of cell-junction genes after *N. meningitidis* infection.

Previous studies with immortalized BECs grown in monoculture showed that *N. meningitidis* modulates the localization of adherens junction proteins together with the polarity complex and affects tight junction expression in brain endothelial cells [25, 28]. To assess the effects of *N. meningitidis* on the localization and expression of occludin, which was not transcriptionally regulated, we analyzed BEC layers of our iBEC-LMC co-culture model after 24 and 30 h of infection with strain MC58 using confocal microscopy and the recently developed junction analyzer software JAnaP [58]. Here, we observed reduced junction coverage 24 h and 30 h p.i. (Fig. 4c). In summary, these results demonstrate that *N. meningitidis* leads to barrier disintegration in BECs at late time point of infection, although barrier function of BECs was enhanced by LMCs in the mBCSFB model.

### Immune response of BECs to N. meningitidis infection with LMC co-culture

**Fig. 5:**
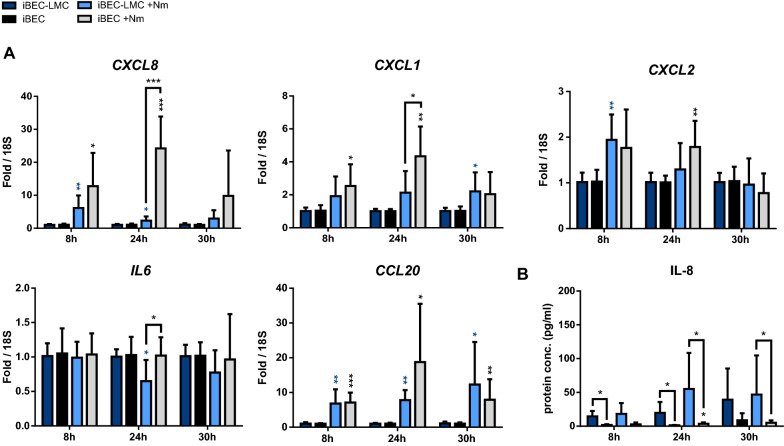
Effects of *N. meningitidis* infection on expression of proinflammatory cytokines in iBECs of mono- and co-culture models. **a** Relative expression of *CXCL8*, *CXCL1*, *CXCL2*, *CCL20*, and *IL6* transcripts in iBECs from direct co-culture with LMCs with (light bars) or without (dark bars) *N. meningitidis* infection, quantified by qPCR and normalized to 18S rRNA. **b** Concentration of IL-8 in the cell culture medium, determined using ELISA. Data presented as mean ± SD from four independent experiments and iBEC differentiations performed in duplicate (n = 6–8). *p < 0.05, **p < 0.01, ***p < 0.001; Student's t test; infected vs. uninfected control (blue/black asterisks directly over bars), mono vs co-culture (asterisks above brackets)

Finally, we examined immune activation of BECs in our mBCSFB models in response to *N. meningitidis* infection, considering that BEC activation presumably contributes to recruitment of leukocytes into the CSF and progression of bacterial meningitis [73]. Using qPCR, we investigated gene expression of neutrophilic chemoattractants and activators IL8 (*CXCL8*), C-X-C motif chemokine 1 and 2 (*CXCL1*, *CXCL2*), C–C motif chemokine 20 (*CCL20*), and the broad cytokine Interleukin-6 (*IL6*). In both iBEC-LMC and iBEC monocultures, *CXCL8*, *CXCL1*, *CXCL2*, and *CCL20* were upregulated during the time course of infection, with higher upregulation of *CXCL8* and *CXCL1* in the monoculture model at 24 h p.i. (Fig. 5a). Despite significant increases in mRNA expression, IL-8 was barely detectable by ELISA in the cell culture supernatants (Fig. 5b). Slightly higher protein concentrations were measured in the apical medium of iBECs co-cultured with LMCs. However, this effect may be due to IL-8 secretion by the meningioma derived LMCs, which has previously been described [34]. These observations, including the disconnect between cytokine expression and secretion in iBECs are consistent with previous studies that have used iBECs cultured as monolayers in cell culture plates to model infection with *N. meningitidis* and Group B *Streptococcus* [33, 50]. Highly significant upregulation of all cytokines and chemokines examined, including *CXCL8*, *CXCL1*, *CXCL2*, *CCL20*, as well as *IL6*, was observed in hCMEC/D3 co-culture with LMC (Additional file 2: Fig. S7a). This effect was consistent with data from hMCEC/D3 monoculture, although the relative fold change of *CXCL8*, *CXCL1*, and *IL6* expression was a bit lower at certain time points. In contrast to the iBEC models, high protein levels of IL-8 were detected in the cell-culture supernatants of the hCMEC/D3 models (Additional file 2: Fig. S7b). In summary, these results demonstrate that relevant proinflammatory cytokines are upregulated in iBECs and hCMEC/D3s co-cultured with LMCs, comparable to iBEC and hCMEC/D3 monocultures, and suggest that the molecular response of BECs to *N. meningitidis* infection plays a role in immune activation at the mBSCFB, as previously described [33, 50, 73].

## Discussion

Bacterial meningitis is a severe disease that occurs when pathogens such as *Neisseria meningitidis* (the meningococcus) cross the meningeal blood-cerebrospinal fluid barrier (mBCSFB) and infect the meninges [1, 2]. The mBCSFB consists of specialized brain endothelial cells (BECs) that exhibit a barrier phenotype to maintain brain homeostasis and are surrounded by leptomeningeal cells (LMCs) [1, 8]. Due to the human-exclusive tropism of *N. meningitidis*, most studies examining meningococcal interaction at the mBCSFB have utilized primary or immortalized BECs that, however, lack critical barrier phenotypes in vitro [11, 40, 41]. *N. meningitidis* interaction with LMCs alone has been investigated using LMCs derived from meningioma [34–37]. However, meningococcal penetration of the mBCSFB has not yet been studied in a multicellular context including this cell type in vitro. Here, we used iPSC derived BECs (iBECs) or hCMEC/D3 cells in co-culture with meningioma-derived LMCs to develop a more complex and physiologically relevant in vitro model for studying *N. meningitidis*-mBCSFB interaction.

Hallmark phenotypes of BECs include endothelial markers, tight junction expression, barrier properties, functional nutrient and efflux transporters, and response to other CNS cell types [74]. To benchmark and validate BEC in vitro models, certain methods including TEER measurements, permeability assays, and immunostainings of key markers such as endothelial adherens and tight junctions are used [40]. Primary and immortalized BECs are scalable and have been widely used for modeling of blood-CNS barriers but frequently lose important barrier phenotypes once removed from their native microenvironment [11]. The extensively characterized immortalized microvascular endothelial cell line hCMEC/D3 retains many BEC characteristics but exhibits low TEER and often lacks continuous expression of tight junction components at the cell–cell junctions [11, 41–43, 75] (Additional file 2: Fig. S2). Despite these drawbacks, hCMEC/D3s are a robust and widely used in vitro model to study *N. meningitidis*-BEC interaction [14–16, 28].

Advances in stem-cell technologies have generated model brain endothelial-like cells derived from iPSCs, which possess all relevant BEC phenotypes including endothelial markers, tight junction expression, barrier properties, response to other CNS cell types, and functional efflux transporters [11, 44, 45, 48, 49, 66]. Following the publication of the initial protocols, much research has been conducted on these models, uncovering advantages as well as weaknesses, and establishing alternative protocols and improvements. Described limitations are the expression of epithelial genes and proteins, which has been detected in addition to the endothelial phenotypes described [76]. In this study, we differentiated iBECs from iPSC line IMR90-4 according to previously published protocols [44, 46, 48] and co-cultured them with LMCs on permeable transwell inserts. We observed clear expression of important brain endothelial markers such as CD31, VE-cadherin, and claudin-5 within the iBEC layer, but epithelial characteristics were also present, particularly related to the cytoarchitecture, such as small protrusions on the apical surface, larger cell height and nucleus, and epithelial-like organization of intercellular junctions (Fig. 2). Although these limitations must be considered when employing the model, it remains suitable for certain applications due to its main advantages such as the tight barrier properties. Recently, iBECs have been useful for modeling various diseases of the CNS including Huntington’s disease, MCT8 deficiency (causing Allan-Hurndon-Dudley syndrome), and infectious disease [33, 46, 50–53, 77–79].

Important benchmarks for the barrier properties of in vitro BBB models are high TEER inversely related to paracellular permeability of solutes (although non-linear) [40], although in vivo data are are only available for pial microvessels in anesthetized frogs and rats [80]. This relationship (one-phase exponential decay) was first demonstrated on rat primary BECs, where permeability coefficient values for sodium fluorescein were below 2 × 10^–6^ cm/s above a threshold TEER of 130 Ω cm^2^ [81]. iPSC derived BECs typically reach TEER values above 1500 Ω x cm^2^ and NaF permeability values in the order of 10^–7^ cm/s [33, 44, 46, 47, 49, 66] (Fig. 2c,d). In this study, we observed that LMC co-culture further increased iBEC barrier tightness and stability over multiple days as reflected by lower NaF permeability and higher TEER. While iBECs alone can exhibit high paracellular tightness, co-culture with other cells from the neurovascular unit such as astrocytes and pericytes has been reported to assert stimulating as well as stabilizing effects on iBEC barrier properties, demonstrating that iBECs respond to cues from other CNS cell types [11, 40, 44, 45, 49, 66, 82–84]. Astrocytes and pericytes have also been shown to induce BBB properties in primary bovine, porcine, rodent, or primate BEC models, which exhibited physiologically relevant levels of paracellular restriction that human primary and immortalized BEC lines did not reach [11, 40, 41, 85]. Co-culture with leptomeningeal cells, which are important in the context of the human mBCSFB, has not been explored before. We observed slightly higher TEER and lower NaF permeability of hCMEC/D3 cells co-cultured directly on the transwell membrane with LMCs compared to monoculture, although these effects could not be correlated with changes in barrier phenotype of hCMEC/D3 layers due to the low overall TEER.

Recognizing the advantage of a human in vitro system exhibiting physiological barrier tightness, an increasing number of studies has recently used iPSC derived BECs to model interaction with CNS pathogens such as GBS, Zika virus, and more recently SARS-CoV2, particularly to investigate how such pathogens affect and penetrate the blood-CNS barriers [33, 46, 50–54]. Recently, we validated iPSC derived BECs for infection studies with the human-specific bacterium *N. meningitidis* [33]. As monoculture in vitro models only distantly represent the native microenvironment and the use of in vivo models to study interaction with human-specific *N. meningitidis* is limited to humanized rodents [86], more complex in vitro models could be useful to study this host–pathogen interaction. Co-culture systems with iBECs and other CNS cell types are now widely used to model function and dysfunction of blood-CNS barriers [40, 44, 45, 49, 66, 82–84] but have not been used for infection studies with CNS pathogens yet. Therefore, we developed and used the iBEC-LMC co-culture model to examine meningococcal interaction with and traversal of the mBCSFB.

We observe substantial bacterial adherence to iBECs in our iBEC-LMC co-culture system soon after infection, consistent with our results using hCMEC/D3s with LMC co-culture as well as published data [14, 17, 18, 26]. This tight interaction, which is primarily mediated by meningococcal type IV pili (Tfp), is critical for vascular colonization and, ultimately, penetration of the mBCSFB [14–18, 26, 28]. The mechanism of barrier traversal by *N. meningitidis* has been a matter of debate for a long time. Most bacteria that can cause meningitis including *N. meningitidis*, Group B *Streptococcus*, *Streptococcus pneumoniae*, and *Escherichia coli* K1, penetrate blood-CNS barriers such as the mBCSFB via a transcellular pathway following cellular invasion by the bacteria or via a paracellular pathway that becomes available through disruption of cellular junctions or cell damage [1, 2]. Cellular invasion of BECs by *N. meningitidis* has been observed using peripheral, bone marrow derived, brain microvessel derived, and, recently, also iPSC derived BECs in vitro, suggesting a transcellular route for *N. meningitidis* traversal of the mBCSFB [17, 18, 26, 27, 29–33]. In this study, we also found that *N. meningitidis* invades iPSC derived BECs and hCMEC/D3 cells in our newly developed co-culture systems with LMCs. While relatively low at first, bacterial invasion increased significantly during prolonged infection, which was previously reported in another immortalized BEC model and potentially results from the demasking of adhesins and invasions upon downregulation of the polysaccharide capsule [18]. Co-culture with LMCs did not affect *N. meningitidis* adherence or invasion of BECs in our assays. Finally, we observed increasing meningococcal transmigration of our iBEC and iBEC-LMC models within 24 h of infection while barrier integrity was still very high. Considering that these models exhibit physiological barrier tightness, these observations further support the hypothesis of transcellular barrier traversal by *N. meningitidis*.

Interestingly, we observed higher rates of bacterial transmigration often correlated with lower TEER. For instance, compared to data from the iBEC models, transmigration rates were already substantially higher in the hCMEC/D3 based models at the earliest measured time point after infection, and we even detected more meningococci traversing the iBEC monoculture than the iBEC-LMC co-culture model early on, although absolute counts were low. This suggests that using models exhibiting physiological barrier tightness may be important for studying bacterial traversal, and it indicates that *N. meningitidis* likely crosses the BEC barrier via a paracellular route if available. Previous studies have suggested that this pathway does become available through disruption of cellular junctions [15, 25, 28, 33]. Increased permeability to lucifer yellow and discontinuous junctional localization of adherens junction protein VE-Cadherin was detected and correlated with meningococcal BEC barrier traversal in infected hCMEC/D3s [15, 28]. Mechanistically, junctional disorganization was caused by signaling events triggered by Tfp mediated interaction between *N. meningitidis* and hCMEC/D3s that lead to the recruitment of cytoskeletal and cell-junction components underneath adherent meningococcal colonies [15, 28]. Using another immortalized BEC cell line (HBMEC), cleavage of tight junction protein occludin and cell detachment mediated by matrix-metalloproteinase MMP-8 was observed upon prolonged *N. meningitidis* challenge [25]. In this study, we observed loss of TEER and reduced junction coverage of occludin in iBECs co-cultured with LMCs after 24 h of infection. Additionally, previous data from iBEC monocultures grown on plate indicates cleavage of occludin induced by *N. meningitidis* infection [33]. Together, this suggests modulation of occludin in infected iBECs, although further investigation is required to fully elucidate this mechanism.

In addition to disorganization of cell-junction components, we examined effects of *N. meningitidis* infection on gene expression of adherens and tight junction proteins in BECs as a potential mechanism for barrier deterioration and found that expression of genes coding for VE-Cadherin, ZO-1 and especially endothelial specific tight junction protein claudin-5 was significantly downregulated in infected iBECs, predominantly after 24 h of infection or later. Simultaneously, Snail-1 (*SNAI1*), a transcriptional repressor of tight junctions [67, 68, 70, 71], previously linked to Group B *Streptococcus*, *Streptococcus pneumoniae*, and *E. coli* K1 induced BBB disruption [69, 87, 88], was upregulated. These results were consistent between iBEC-LMC co-culture, iBEC monoculture on transwell and iBECs cultured on plate [33]. Furthermore, *N. meningitidis* infection had similar effects on expression of cell-junction genes as well as *SNAI1* in hCMEC/D3s with and without LMC co-culture. Together, these findings suggest downregulation of tight and adherens junction genes in addition to reorganization of junction proteins during *N. meningitidis* induced barrier disruption. However, transcriptional repression mediated by Snail1 seems not to be the sole mechanism behind this effect, as indicated by siRNA knockdown experiments. Further investigation is required to elucidate this mechanism. In conclusion, while meningococcal invasion of BECs potentially contributes to early traversal, deterioration of barrier properties may open up a more accessible paracellular route later on during infection.

Meningococcal interaction with the mBCSFB and proliferation in the subarachnoid space evokes a strong inflammatory response that is triggered by immune activation of BECs and LMCs and leads to the influx of leukocytes, primarily neutrophils at first [1, 8]. Previous studies have shown that Group B *Streptococcus* and *N. meningitidis* elicit upregulation of neutrophilic chemoattractants in iPSC derived BECs [33, 50]. Consistent with these findings, we observed transcriptional upregulation of IL-8 (*CXCL8*), *CXCL1, CXCL2* and *CCL20* in iBECs cultured on transwell inserts with and without LMCs. This demonstrates that iBECs activate innate immune response mechanism in response to bacterial infection. However, despite the transcriptional upregulation of these factors, secretion of the related proteins was almost undetectable as our study as well as previous investigations have shown [33, 50]. hCMEC/D3s responded to the infection in similar fashion, although all analyzed gene transcripts were upregulated much more substantially and high levels of cytokine secretion were detected in this model. Further investigation is required to fully elucidate the inflammatory response of BECs to bacterial infection and determine if the low abundance of secreted cytokines observed in iBECs is biologically relevant. Activation of LMCs upon meningococcal interaction following mBCSFB traversal likely contributes to inflammation in the subarachnoid spaces, too, and secretion of various cytokines after *N. meningitidis* infection has been demonstrated in meningioma derived LMCs [34, 35, 37].

## Conclusions

In this study, we report, for the first time, co-culture of human iPSC derived BECs or hCMEC/D3s with meningioma derived LMCs to study *N. meningitidis* interaction at the mBCSFB in a physiologically relevant context. As described for co-culture with other CNS cell types, iBECs respond to cues from co-cultured LMCs, which leads to improvement in barrier tightness and stability. *N. meningitidis* interacts with and penetrates iBEC-LMC co-culture models, and disrupts barrier function, consistent with previous data. BEC response to infection was generally not affected by LMC co-culture. Overall, our data suggests that models exhibiting physiological barrier tightness can provide relevant insight into modulation and penetration of blood-CNS barriers by pathogens, demonstrating the usefulness of iBECs for modeling interaction with the meningeal pathogen *N. meningitidis*.

## Supplementary Information

**Additional file 1: Video S1.** Immunofluorescence staining of iBEC-LMC co-culture for tight junction protein ZO-1, performed after 2 days of co-culture (animated volume view). Nucleus staining with DAPI (blue).

**Additional file 2: Figure S1.** Laminin expression in the iBEC-LMC co-culture model. **Figure S2.** Characterization of the hCMEC/D3-LMC co-culture model. **Figure S3.** Barrier properties of LMC monolayers and prolonged iBEC-LMC co-culture. **Figure S4.** *N. meningitidis* interaction with the hCMEC/D3-LMC direct co-culture model. **Figure S5.** Effects of *N. meningitidis* infection on cell-junction expression in hCMEC/D3 from mono- and co-culture models. **Figure S6.** Effects of *SNAI1* knockdown on *N. meningitidis* induced downregulation of cell-junction expression in hCMEC/D3. **Figure S7.** Effects of *N. meningitidis* infection on the expression of proinflammatory cytokines in hCMEC/D3 from mono- and co-culture models.

## Data Availability

The datasets used and/or analyzed during the current study are available from the corresponding author on reasonable request.

## Abbreviations

CSF: Cerebrospinal fluid
mBCSFB: Meningeal blood-CSF barrier
CNS: Central nervous system
BBB: Blood–brain barrier
BEC: Brain endothelial cell
LMC: Leptomeningeal cell
iPSC: Induced pluripotent stem cell
iBEC: IPSC-derived BEC
TEM: Transmission electron microscopy
SIM: Structured illumination microscopy
TEER: Transendothelial electrical resistance
NaF: Sodium fluorescein
*Nm*: *Neisseria meningitidis*
MOI: Multiplicity of infection
CFU: Colony forming units
p.i.: Post-infection

## References

[1] Doran KS (2016). Host-pathogen interactions in bacterial meningitis. Acta Neuropathol.

[2] Le Guennec L (2020). Strategies used by bacterial pathogens to cross the blood-brain barrier. Cell Microbiol.

[3] Jafri RZ (2013). Global epidemiology of invasive meningococcal disease. Popul Health Metr.

[4] Rouphael NG, Stephens DS (2012). Neisseria meningitidis: biology, microbiology, and epidemiology. Methods Mol Biol.

[5] Stephens DS, Greenwood B, Brandtzaeg P (2007). Epidemic meningitis, meningococcaemia, and Neisseria meningitidis. Lancet.

[6] Caugant DA, Maiden MC (2009). Meningococcal carriage and disease–population biology and evolution. Vaccine.

[7] Brandtzaeg P, van Deuren M (2012). Classification and pathogenesis of meningococcal infections. Methods Mol Biol.

[8] Weller RO (2018). The meninges as barriers and facilitators for the movement of fluid, cells and pathogens related to the rodent and human CNS. Acta Neuropathol.

[9] Coureuil M (2017). A journey into the brain: insight into how bacterial pathogens cross blood-brain barriers. Nat Rev Microbiol.

[10] Engelhardt B, Sorokin L (2009). The blood-brain and the blood-cerebrospinal fluid barriers: function and dysfunction. Semin Immunopathol.

[11] Helms HC (2016). In vitro models of the blood-brain barrier: an overview of commonly used brain endothelial cell culture models and guidelines for their use. J Cereb Blood Flow Metab.

[12] Rua R, McGavern DB (2018). Advances in meningeal immunity. Trends Mol Med.

[13] Schubert-Schubert A (2017). Molecular mechanisms involved in the interaction of Neisseria meningitidis with cells of the human blood-cerebrospinal fluid barrier. Pathog Dis.

[14] Bernard SC (2014). Pathogenic neisseria meningitidis utilizes CD147 for vascular colonization. Nat Med.

[15] Coureuil M (2010). Meningococcus Hijacks a beta2-adrenoceptor/beta-arrestin pathway to cross brain microvasculature endothelium. Cell.

[16] Maissa N (2017). Strength of Neisseria meningitidis binding to endothelial cells requires highly-ordered CD147/beta2-adrenoceptor clusters assembled by alpha-actinin-4. Nat Commun.

[17] Sa ECC, Griffiths NJ, Virji M (2010). Neisseria meningitidis Opc invasin binds to the sulphated tyrosines of activated vitronectin to attach to and invade human brain endothelial cells. PLoS Pathog.

[18] Unkmeir A (2002). Fibronectin mediates Opc-dependent internalization of Neisseria meningitidis in human brain microvascular endothelial cells. Mol Microbiol.

[19] Comanducci M (2002). NadA, a novel vaccine candidate of Neisseria meningitidis. J Exp Med.

[20] Hung MC, Christodoulides M (2013). The biology of neisseria adhesins. Biology.

[21] Scarselli M (2006). Neisseria meningitidis NhhA is a multifunctional trimeric autotransporter adhesin. Mol Microbiol.

[22] Turner DP (2006). Characterization of MspA, an immunogenic autotransporter protein that mediates adhesion to epithelial and endothelial cells in Neisseria meningitidis. Infect Immun.

[23] Peters S (2021). A comprehensive review on the interplay between Neisseria spp. and host sphingolipid metabolites. Cells.

[24] Peters S (2019). Neisseria meningitidis type IV Pili Trigger Ca(2+)-dependent lysosomal trafficking of the acid sphingomyelinase to enhance surface ceramide levels. Infect Immun..

[25] Schubert-Unkmeir A (2010). Neisseria meningitidis induces brain microvascular endothelial cell detachment from the matrix and cleavage of occludin: a role for MMP-8. PLoS Pathog.

[26] Simonis A (2014). Differential activation of acid sphingomyelinase and ceramide release determines invasiveness of Neisseria meningitidis into brain endothelial cells. PLoS Pathog.

[27] Slanina H (2012). Cell invasion by Neisseria meningitidis requires a functional interplay between the focal adhesion kinase, Src and cortactin. PLoS ONE.

[28] Coureuil M (2009). Meningococcal type IV pili recruit the polarity complex to cross the brain endothelium. Science.

[29] Eugene E (2002). Microvilli-like structures are associated with the internalization of virulent capsulated Neisseria meningitidis into vascular endothelial cells. J Cell Sci.

[30] Lambotin M (2005). Invasion of endothelial cells by Neisseria meningitidis requires cortactin recruitment by a phosphoinositide-3-kinase/Rac1 signalling pathway triggered by the lipo-oligosaccharide. J Cell Sci.

[31] Nikulin J (2006). Intracellular survival and replication of Neisseria meningitidis in human brain microvascular endothelial cells. Int J Med Microbiol.

[32] Sokolova O (2004). Interaction of Neisseria meningitidis with human brain microvascular endothelial cells: role of MAP- and tyrosine kinases in invasion and inflammatory cytokine release. Cell Microbiol.

[33] Martins Gomes SF (2019). Induced pluripotent stem cell-derived brain endothelial cells as a cellular model to study neisseria meningitidis infection. Front Microbiol.

[34] Christodoulides M (2002). Interaction of Neisseria meningitidis with human meningeal cells induces the secretion of a distinct group of chemotactic, proinflammatory, and growth-factor cytokines. Infect Immun.

[35] Fowler MI (2004). Different meningitis-causing bacteria induce distinct inflammatory responses on interaction with cells of the human meninges. Cell Microbiol.

[36] Hardy SJ (2000). Interactions of Neisseria meningitidis with cells of the human meninges. Mol Microbiol.

[37] Humphries HE (2005). Activation of human meningeal cells is modulated by lipopolysaccharide (LPS) and non-LPS components of Neisseria meningitidis and is independent of Toll-like receptor (TLR)4 and TLR2 signalling. Cell Microbiol.

[38] Alkuwaity K (2012). Group B Streptococcus interactions with human meningeal cells and astrocytes in vitro. PLoS ONE.

[39] Auger JP (2015). Interactions of Streptococcus suis serotype 2 with human meningeal cells and astrocytes. BMC Res Notes.

[40] DeStefano JG (2018). Benchmarking in vitro tissue-engineered blood-brain barrier models. Fluids Barriers CNS.

[41] Eigenmann DE (2013). Comparative study of four immortalized human brain capillary endothelial cell lines, hCMEC/D3, hBMEC, TY10, and BB19, and optimization of culture conditions, for an in vitro blood-brain barrier model for drug permeability studies. Fluids Barriers CNS.

[42] Weksler B, Romero IA, Couraud PO (2013). The hCMEC/D3 cell line as a model of the human blood brain barrier. Fluids Barriers CNS.

[43] Weksler BB (2005). Blood-brain barrier-specific properties of a human adult brain endothelial cell line. FASEB J.

[44] Lippmann ES (2014). A retinoic acid-enhanced, multicellular human blood-brain barrier model derived from stem cell sources. Sci Rep.

[45] Lippmann ES (2012). Derivation of blood-brain barrier endothelial cells from human pluripotent stem cells. Nat Biotechnol.

[46] Endres LM, Schubert-Unkmeir A, Kim BJ (2020). Neisseria meningitidis Infection of induced pluripotent stem-cell derived brain endothelial cells. J Vis Exp.

[47] Neal EH (2019). A simplified, fully defined differentiation scheme for producing blood-brain barrier endothelial cells from human iPSCs. Stem Cell Reports.

[48] Stebbins MJ (2016). Differentiation and characterization of human pluripotent stem cell-derived brain microvascular endothelial cells. Methods.

[49] Jamieson JJ (2019). Role of iPSC-derived pericytes on barrier function of iPSC-derived brain microvascular endothelial cells in 2D and 3D. Fluids Barriers CNS.

[50] Kim BJ (2017). Modeling group B streptococcus and blood-brain barrier interaction by using induced pluripotent stem cell-derived brain endothelial cells. mSphere.

[51] Kim BJ (2019). Streptococcus agalactiae disrupts P-glycoprotein function in brain endothelial cells. Fluids Barriers CNS.

[52] Espinal ER (2022). Group B streptococcus-induced macropinocytosis contributes to bacterial invasion of brain endothelial cells. Pathogens.

[53] Alimonti JB (2018). Zika virus crosses an in vitro human blood brain barrier model. Fluids Barriers CNS.

[54] Krasemann S (2022). The blood-brain barrier is dysregulated in COVID-19 and serves as a CNS entry route for SARS-CoV-2. Stem Cell Reports.

[55] McGuinness BT (1991). Point mutation in meningococcal por a gene associated with increased endemic disease. Lancet.

[56] Kim BJ, Schubert-Unkmeir A (2019). In vitro models for studying the interaction of neisseria meningitidis with human brain endothelial cells. Methods Mol Biol.

[57] Mikaty G (2009). Extracellular bacterial pathogen induces host cell surface reorganization to resist shear stress. PLoS Pathog.

[58] Gray KM (2019). Quantitative phenotyping of cell-cell junctions to evaluate ZO-1 presentation in brain endothelial cells. Ann Biomed Eng.

[59] Prufert K, Vogel A, Krohne G (2004). The lamin CxxM motif promotes nuclear membrane growth. J Cell Sci.

[60] Livak KJ, Schmittgen TD (2001). Analysis of relative gene expression data using real-time quantitative PCR and the 2(-Delta Delta C(T)) Method. Methods.

[61] Rho HW (2010). Identification of valid reference genes for gene expression studies of human stomach cancer by reverse transcription-qPCR. BMC Cancer.

[62] Han SP (2011). SNAI1 is involved in the proliferation and migration of glioblastoma cells. Cell Mol Neurobiol.

[63] van Sorge NM (2008). Anthrax toxins inhibit neutrophil signaling pathways in brain endothelium and contribute to the pathogenesis of meningitis. PLoS ONE.

[64] Derk J (2021). Living on the edge of the CNS: meninges cell diversity in health and disease. Front Cell Neurosci.

[65] Halder SK, Sapkota A, Milner R (2022). The impact of genetic manipulation of laminin and integrins at the blood-brain barrier. Fluids Barriers CNS.

[66] Hollmann EK (2017). Accelerated differentiation of human induced pluripotent stem cells to blood-brain barrier endothelial cells. Fluids Barriers CNS.

[67] Carrozzino F (2005). Inducible expression of snail selectively increases paracellular ion permeability and differentially modulates tight junction proteins. Am J Physiol Cell Physiol.

[68] Ikenouchi J (2003). Regulation of tight junctions during the epithelium-mesenchyme transition: direct repression of the gene expression of claudins/occludin by snail. J Cell Sci.

[69] Kim BJ (2015). Bacterial induction of snail1 contributes to blood-brain barrier disruption. J Clin Invest.

[70] Martinez-Estrada OM (2006). The transcription factors Slug and Snail act as repressors of Claudin-1 expression in epithelial cells. Biochem J.

[71] Ohkubo T, Ozawa M (2004). The transcription factor Snail downregulates the tight junction components independently of E-cadherin downregulation. J Cell Sci.

[72] Kaufhold S, Bonavida B (2014). Central role of Snail1 in the regulation of EMT and resistance in cancer: a target for therapeutic intervention. J Exp Clin Cancer Res.

[73] Tunkel AR, Scheld WM (1993). Pathogenesis and pathophysiology of bacterial meningitis. Clin Microbiol Rev.

[74] Abbott NJ (2010). Structure and function of the blood-brain barrier. Neurobiol Dis.

[75] Gericke B (2020). A face-to-face comparison of claudin-5 transduced human brain endothelial (hCMEC/D3) cells with porcine brain endothelial cells as blood-brain barrier models for drug transport studies. Fluids Barriers CNS.

[76] Lippmann ES (2020). Commentary on human pluripotent stem cell-based blood-brain barrier models. Fluids Barriers CNS.

[77] Kim BJ, Shusta EV, Doran KS (2019). Past and current perspectives in modeling bacteria and blood-brain barrier interactions. Front Microbiol.

[78] Lim RG (2017). Huntington’s disease iPSC-derived brain microvascular endothelial cells reveal WNT-mediated angiogenic and blood-brain barrier deficits. Cell Rep.

[79] Vatine GD (2017). Modeling Psychomotor Retardation using iPSCs from MCT8-Deficient Patients Indicates a Prominent Role for the Blood-Brain Barrier. Cell Stem Cell.

[80] Vigh JP (2021). Transendothelial Electrical Resistance Measurement across the Blood-Brain Barrier: a critical review of methods. Micromachines..

[81] Gaillard PJ, de Boer AG (2000). Relationship between permeability status of the blood-brain barrier and in vitro permeability coefficient of a drug. Eur J Pharm Sci.

[82] Appelt-Menzel A (2017). Establishment of a Human Blood-Brain Barrier Co-culture Model Mimicking the Neurovascular Unit Using Induced Pluri- and Multipotent Stem Cells. Stem Cell Reports.

[83] Canfield SG (2019). An isogenic neurovascular unit model comprised of human induced pluripotent stem cell-derived brain microvascular endothelial cells, pericytes, astrocytes, and neurons. Fluids Barriers CNS.

[84] Canfield SG (2017). An isogenic blood-brain barrier model comprising brain endothelial cells, astrocytes, and neurons derived from human induced pluripotent stem cells. J Neurochem.

[85] Hatherell K (2011). Development of a three-dimensional, all-human in vitro model of the blood-brain barrier using mono-, co-, and tri-cultivation transwell models. J Neurosci Methods.

[86] Johswich KO (2013). In vivo adaptation and persistence of Neisseria meningitidis within the nasopharyngeal mucosa. PLoS Pathog.

[87] Gratz N (2017). Pneumococcal neuraminidase activates TGF-beta signalling. Microbiology.

[88] Yang R (2016). Induction of VEGFA and Snail-1 by meningitic Escherichia coli mediates disruption of the blood-brain barrier. Oncotarget.

