# Supplementary material for: Development of a multicellular in vitro model of the meningeal blood-CSF barrier to study Neisseria meningitidis infection
Source: Fluids Barriers CNS. 2022 Oct 26;19:81. doi: 10.1186/s12987-022-00379-z (PMC9597984; doi:10.1186/s12987-022-00379-z)
Supplement: Supplementary file 2 — Additional file 2: Figure S1. Laminin expression in the iBEC-LMC co-culture model. Figure S2. Characterization of the hCMEC/D3-LMC co-culture model. Figure S3. Barrier properties of LMC monolayers and prolonged iBEC-LMC co-culture. Figure S4. N. meningitidis interaction with the hCMEC/D3-LMC direct co-culture model. Figure S5. Effects of N. meningitidis infection on cell-junction expression in hCMEC/D3 from mono- and co-culture models. Figure S6. Effects of SNAI1 knockdown on N. meningitidis induced downregulation of cell-junction expression in hCMEC/D3. Figure S7. Effects of N. meningitidis infection on the expression of proinflammatory cytokines in hCMEC/D3 from mono- and co-culture models. [file 12987_2022_379_MOESM2_ESM.pdf]

Figure S1

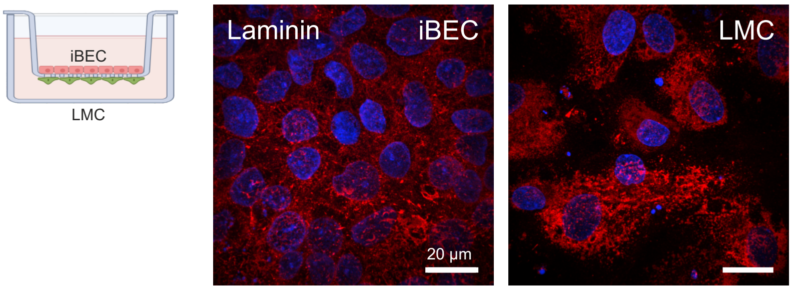

Fig. S1 Laminin expression in the iBEC-LMC co-culture model. Pan-Laminin immunofluorescence staining (red) of iBEC and LMC monolayers on either side of transwell membranes, performed after 2 days of co-culture. Nucleus staining with DAPI (blue). Scale bars represent 20 µm.

Figure S2

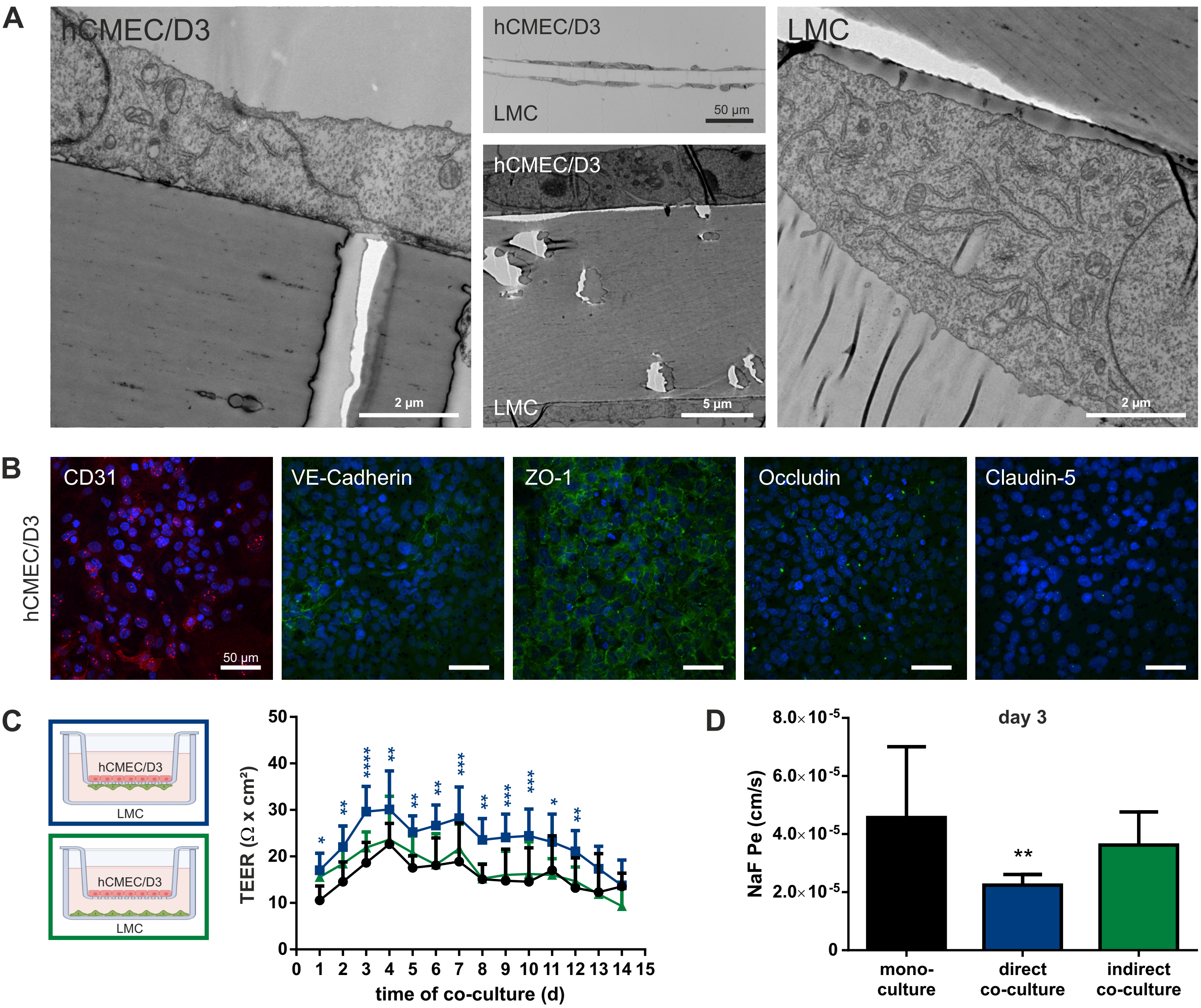

Fig. S2 Characterization of the hCMEC/D3-LMC co-culture model. **a** Transmission electron microscopy of hCMEC/D3 and LMCs co-cultured on transwell for 3 days. A widefield image of a semithin cross-section of the embedded model (middle, top) is presented in addition to electron micrographs of ultrathin sections. **b** Immunofluorescence staining of the hCMEC/D3 layer for endothelial adherence junction proteins (CD31 and VE-Cadherin) and tight junction components (ZO-1, Occludin, and claudin-5), performed after 3 days of co-culture. Nucleus staining with DAPI (blue). Scale bars represent 50 µm. **c** TEER values were measured each day in hCMEC/D3 monoculture (black), direct (blue) and indirect (green) or hCMEC/D3-LMC co-culture over a time-course of 14 days. **d** NaF Pe of hCMEC/D3 mono and co-culture models on day 3 of co-culture. All data presented as mean ± SD from three independent experiments performed in triplicate (n = 9). *$p < 0.05$, **$p < 0.01$, ***$p < 0.001$, **** $p < 0.0001$; ANOVA followed by Dunnett's multiple comparisons test; direct (blue) and indirect (green) co-culture vs monoculture.

Figure S3

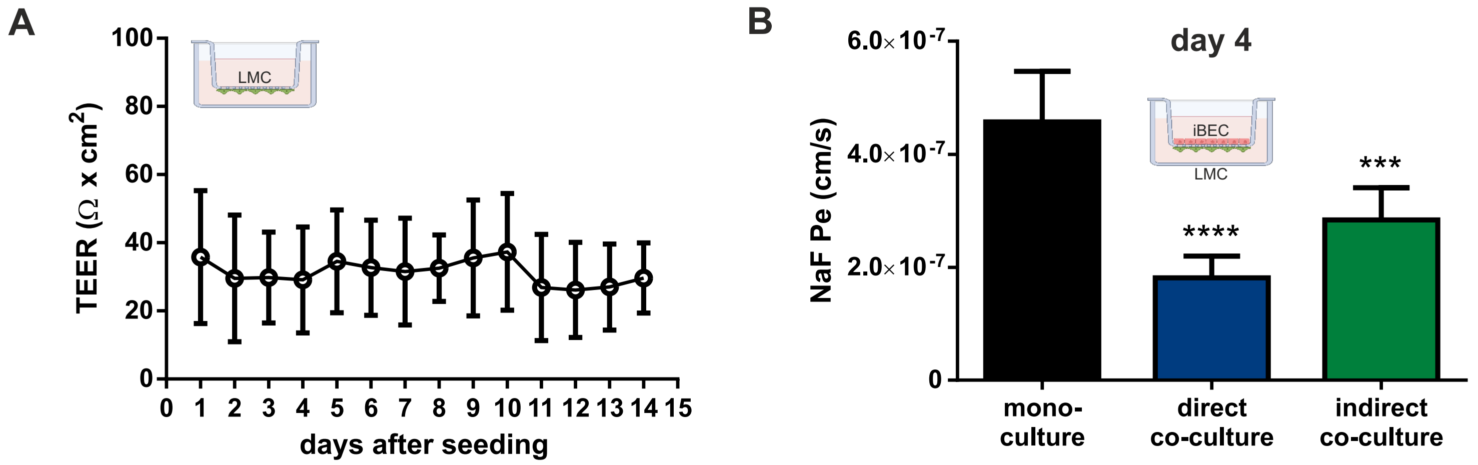

Fig. S3 Barrier properties of LMC monolayers and prolonged iBEC-LMC co-culture. **a** TEER of LMC monolayers cultured on the underside of transwell inserts and measured daily over a time-course of 14 days. Data from three independent experiments performed in triplicate (n = 9). **b** Sodium fluorescein permeability (NaF Pe) of iBEC monoculture (black), direct (blue) and indirect (green) iBEC-LMC co-culture on day 4 of co-culture. Data from two independent experiments performed in triplicate (n = 6). All data presented as mean ± SD. ***p < 0.001, **** p < 0.0001; ANOVA followed by Dunnett's multiple comparisons test; direct (blue) and indirect (green) co-culture vs monoculture.

Figure S4

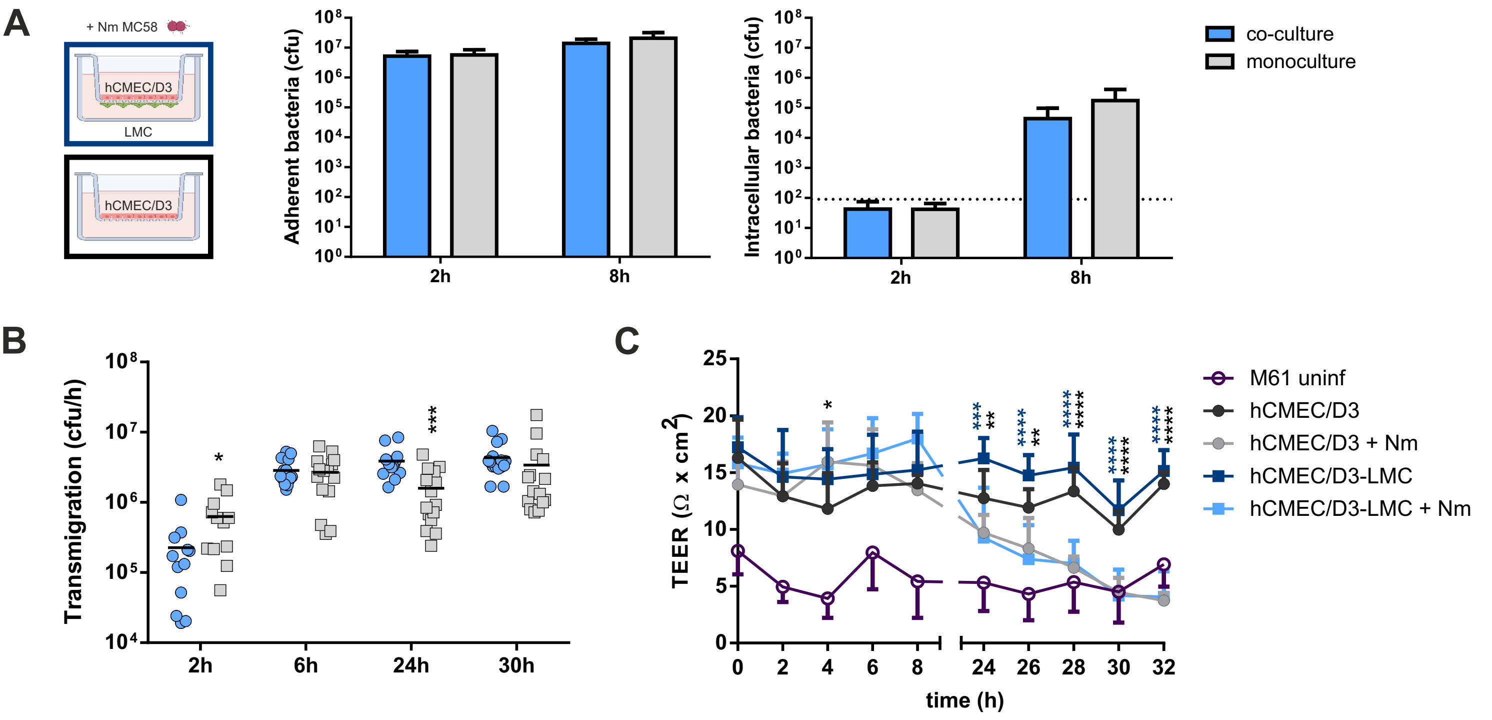

Fig. S4 *N. meningitidis* interaction with the hCMEC/D3-LMC direct co-culture model. hCMEC/D3 were infected from the apical side (MOI = 10, unless specified otherwise) on day 3 of co-culture for the times indicated. **a** Enumeration of adherent and intracellular cfu/monolayer on hCMEC/D3 layers with or without LMC co-culture after infection with *N. meningitidis* at an MOI of 100, determined by gentamicin protection assays. Data from four independent experiments performed in duplicate (n = 8). **b** *N. meningitidis* transmigration rates determined by enumeration of cfu in the basolateral compartment after 1 h of incubation in fresh basolateral media following the indicated infection time points. **c** TEER values of infected and uninfected hCMEC/D3 monoculture and hCMEC/D3-LMC co-culture over a time-course of 32 h. Data from three independent experiments performed in triplicate (n = 9). Data presented as mean ± SD. *p < 0.05, **p < 0.01, ***p < 0.001, ****p < 0.0001; Student's t test; direct co-culture (blue) vs monoculture (grey) (**a, b**), infected vs uninfected control (**c**).

Figure S5

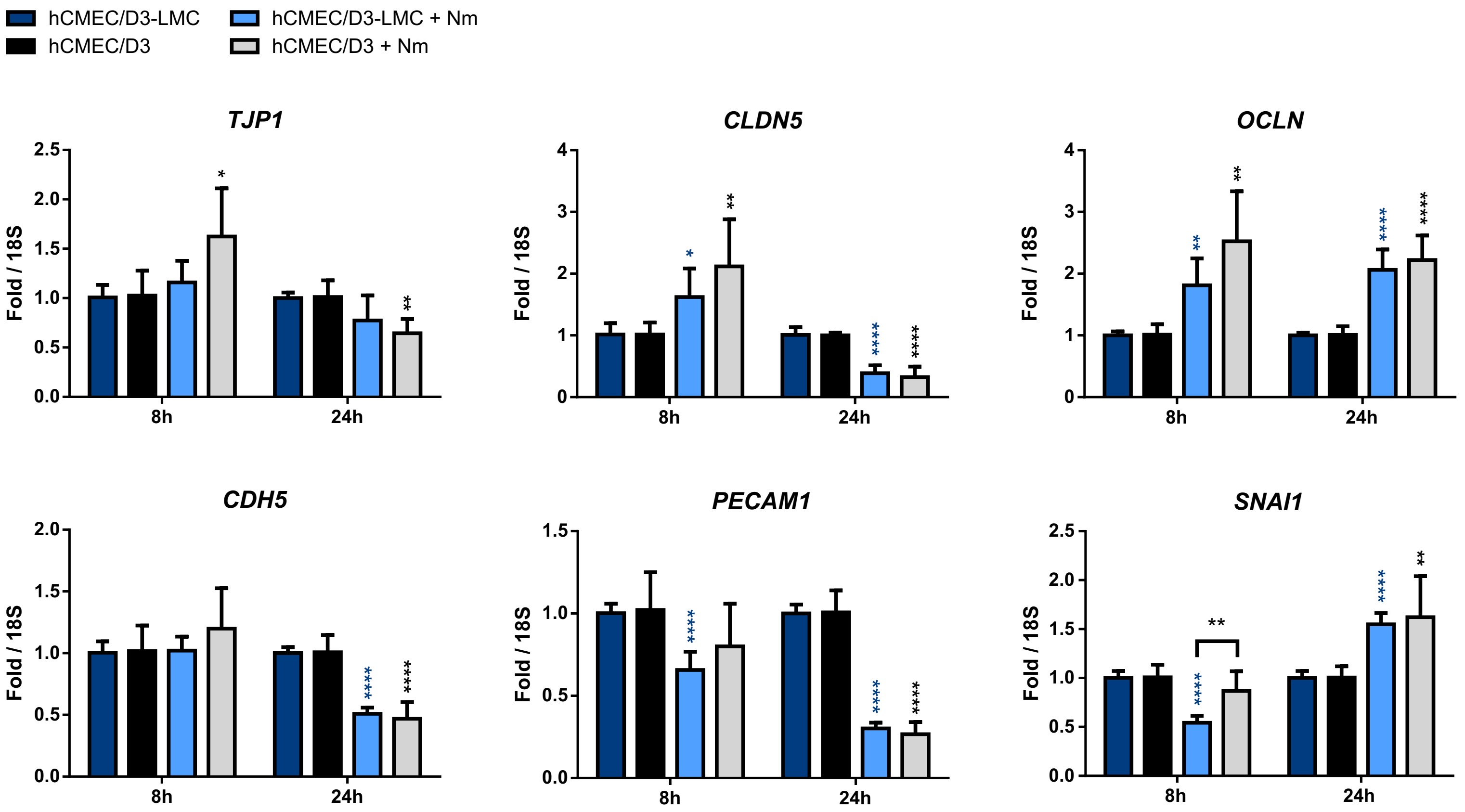

Fig. S5 Effects of *N. meningitidis* infection on cell-junction expression in hCMEC/D3 from mono- and co-culture models. Relative expression of genes for endothelial adherence junction proteins CD31 (*PECAM1*) and VE-Cadherin (*CDH5*), and tight junction components ZO-1 (*TJP1*), Occludin (*OCLN*), and claudin-5 (*CLDN5*) in hCMEC/D3 from direct co-culture with LMCs (blue bars) and hCMEC/D3 transwell monoculture (black/gray bars) with (light bars) or without (dark bars) *N. meningitidis* infection, quantified by qPCR and normalized to 18S rRNA. Data presented as mean ± SD from three independent experiments performed in duplicate (n = 6). *$p < 0.05$, **$p < 0.01$, ****$p < 0.0001$; Student's t test; infected vs. uninfected control (blue/black asterisks directly over bars), mono vs co-culture (asterisks above brackets).

Figure S6

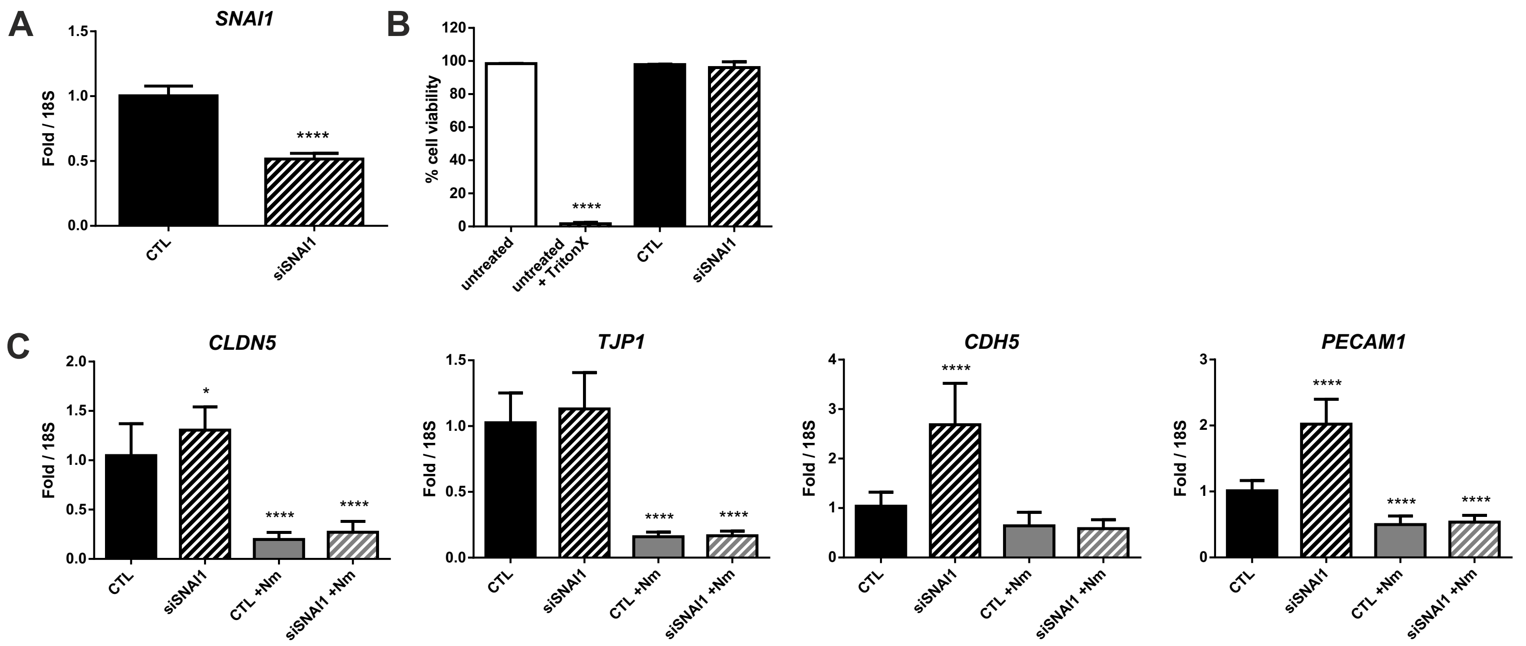

Fig. S6 Effects of *SNAI1* knockdown on *N. meningitidis* induced downregulation of cell-junction expression in hCMEC/D3. **a** Relative expression of **SNAI1** after siRNA mediated knockdown (*siSNAI1*) compared to scrambled control siRNA (*CTL*), quantified by qPCR and normalized to 18S rRNA. **b** Percentage of viable cells after siRNA transfection, determined via PI staining and flow cytometry using untreated hCMEC/D3 as controls. **c** Relative expression of genes for tight junction components ZO-1 (*TJP1*) and claudin-5 (*CLDN5*), and endothelial adherence junction proteins VE-Cadherin (*CDH5*) and CD31 (*PECAM1*) in hCMEC/D3 after siRNA knockdown of *SNAI1* and with (light bars) or without (dark bars) *N. meningitidis* infection, quantified by qPCR and normalized to 18S rRNA. Data presented as mean ± SD from three independent experiments performed in triplicate (n = 9). ****$p < 0.0001$; Student's t test (**a**); ANOVA followed by Dunnett's multiple comparisons test (**b, c**).

Figure S7

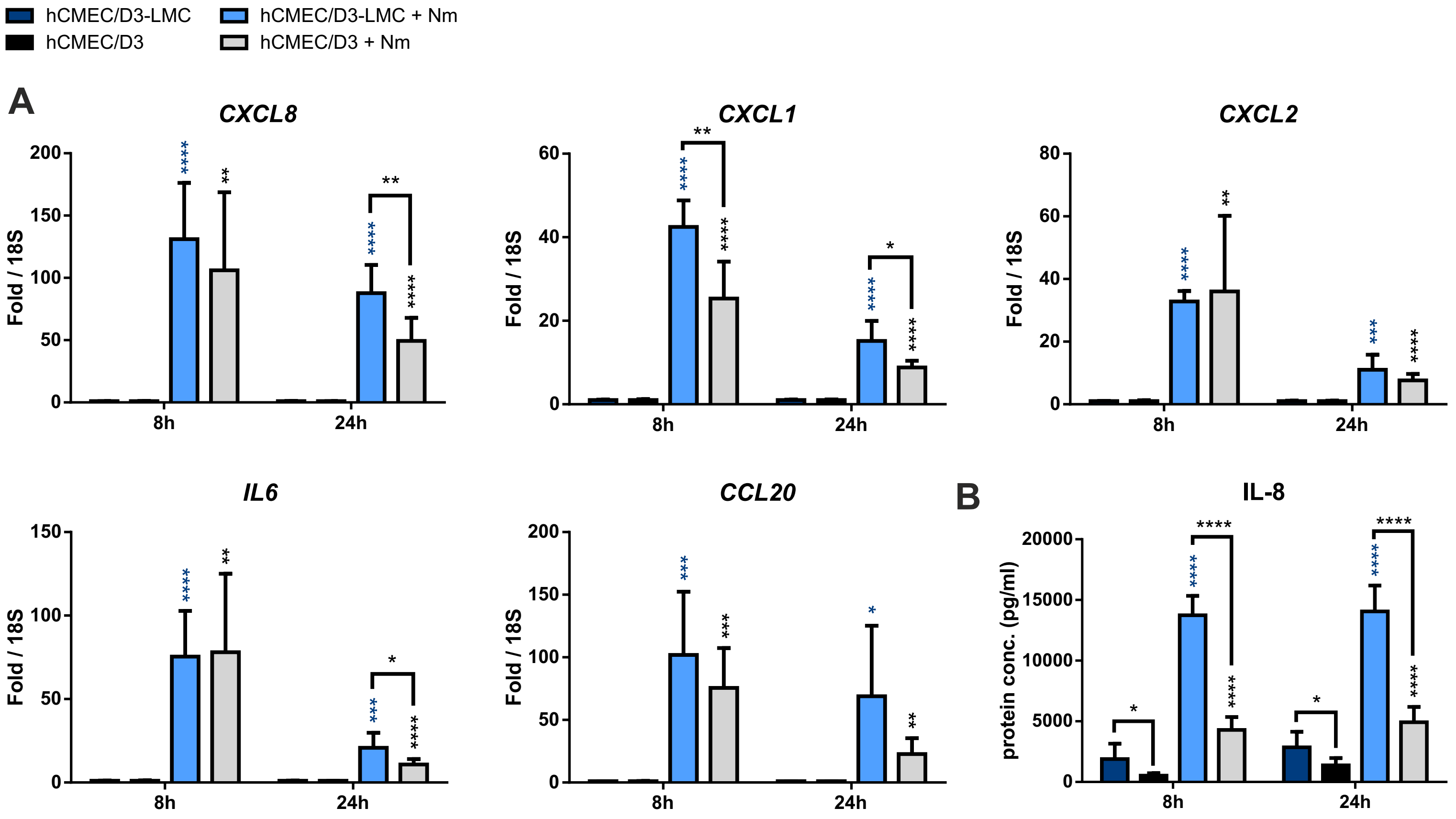

Fig. S7 Effects of *N. meningitidis* infection on the expression of proinflammatory cytokines in hCMEC/D3 from mono- and co-culture models. **a** Relative expression of *CXCL8*, *CXCL1*, *CXCL2*, *CCL20*, and *IL6* transcripts in hCMEC/D3 from direct co-culture with LMCs (blue bars) and hCMEC/D3 transwell monoculture (black/gray bars) with (light bars) or without (dark bars) *N. meningitidis* infection, quantified by qPCR and normalized to 18S rRNA. **b** Concentration of IL-8 in the cell culture medium, determined using ELISA. Data presented as mean ± SD from three independent experiments performed in duplicate (n = 6). *$p < 0.05$, **$p < 0.01$, ***$p < 0.001$, ****$p < 0.0001$; Student's t test; infected vs. uninfected control (blue/black asterisks directly above bars), mono vs co-culture (asterisks above brackets).
